# LncRNA *PVT1* induces mitochondrial dysfunction of podocytes via TRIM56 in diabetic kidney disease

**DOI:** 10.1038/s41419-024-07107-5

**Published:** 2024-09-30

**Authors:** Zhimei Lv, Ziyang Wang, Jinxiu Hu, Hong Su, Bing Liu, Yating Lang, Qun Yu, Yue Liu, Xiaoting Fan, Meilin Yang, Ning Shen, Dongdong Zhang, Xia Zhang, Rong Wang

**Affiliations:** 1 Department of Nephrology, Shandong Provincial Hospital Affiliated to Shandong First Medical University, Jinan, Shandong 250021 China; 2 Department of Nephrology, Shandong Provincial Hospital, Shandong University, Jinan, Shandong 250021 China

**Keywords:** Mechanisms of disease, Diabetic nephropathy

## Abstract

Mitochondrial dysfunction is a significant contributor to podocyte injury in diabetic kidney disease (DKD). While previous studies have shown that *PVT1* might play a vital role in DKD, the precise molecular mechanisms are largely unknown. By analyzing the plasma and kidney tissues of DKD patients, we observed a significant upregulation of *PVT1* expression, which exhibited a positive correlation with albumin/creatinine ratios and serum creatinine levels. Then, we generated mice with podocyte-specific deletion of *PVT1* (*Nphs2-Cre/Pvt1*^*flox/flox*^) and confirmed that the deletion of PVT1 suppressed podocyte mitochondrial dysfunction and inflammation in addition to ameliorating diabetes-induced podocyte injury, glomerulopathy, and proteinuria. Subsequently, we cultured podocytes in vitro and observed that *PVT1* expression was upregulated under hyperglycemic conditions. Mechanistically, we demonstrated that *PVT1* was involved in mitochondrial dysfunction by interacting with TRIM56 post-transcriptionally to modulate the ubiquitination of AMPKα, leading to aberrant mitochondrial biogenesis and fission. Additionally, the release of mtDNA and mtROS from damaged mitochondria triggered inflammation in podocytes. Subsequently, we verified the important role of TRIM56 in vivo by constructing Nphs2-Cre/Trim56^flox/flox^ mice, consistently with the results of *Nphs2-Cre/Pvt1*^*flox/flox*^ mice. Together, our results revealed that upregulation of PVT1 could promote mitochondrial dysfunction and inflammation of podocyte by modulating TRIM56, highlighting a potential novel therapeutic target for DKD.

## Introduction

Diabetic kidney disease (DKD), one of the chronic microvascular complications of diabetes mellitus (DM), is the leading cause of end stage renal disease (ESRD) globally [1, 2]. In recent years, the prevalence of DKD has grown robustly due to more patients with T2DM [3]. Podocyte injury is considered the core event of DKD [4].

Podocytes are highly differentiated cells, with a limited capacity for renewal and require a substantial amount of energy to maintain the complex cellular morphology and structure, which in turn requires maintaining an adequate mitochondrial number and their proper function [5]. Numerous studies have suggested that mitochondrial dysfunction contributes to podocyte injury [6–8]. Hence, it is of great significance to identify the key molecules involved in the mitochondrial dysfunction in podocytes during the progression of DKD.

Long noncoding RNAs (lncRNAs), a heterogeneous group of transcripts with a minimal length of 200 nucleotides, has been proposed to be involved in DKD [9, 10]. LncRNA plasmacytoma variant translocation 1 (*PVT1*), located on chromosome 8q24.21, has been found to be associated with diabetic kidney disease [11]. Hanson et al. performed a genome-wide analysis of 115,352 single nucleotide polymorphisms (SNPs), and explicated that *PVT1* may function as a candidate gene for contributing to ESRD susceptibility in diabetes [12]. Although the association between *PVT1* and kidney lesion has been demonstarted, the precise molecular mechanisms by which PVT1 regulates podocytes injury and whether PVT1 is involved in mitochondrial dysfunction of podocytes during the progression of DKD are largely unknown.

Here, we utilized human samples, cultured human podocytes, and podocyte-specific knockout animal model to study the role and mechanisms of *PVT1* in regulating podocyte mitochondrial function in DKD. Our results revealed a critical role of *PVT1* in DKD pathogenesis and progression, which may offer promising clues to develop new therapeutic strategies for patients with DKD.

## Materials and methods

### Patients and clinical samples

The use of human samples in this research was sanctioned by the Institutional Ethical Review Boards (NSFC: NO.2018-051) and the signed patient informed consent were acquired in chorus. Plasma samples of patients with DKD and healthy controls were collected from Shandong Provincial Hospital from January 2018 to September 2021. In addition, freshly frozen normal kidney samples (>2 cm adjacent to urological neoplasms) and DKD renal biopsy samples were also acquired from the identical hospital between January 2018 and December 2022.

### Production of conditional knockout mouse model

The production of podocyte-specific knockout mice were constructed by Cyagen Biosciences (Suzhou) Inc. In brief, exon 6-8 selected as conditional knockout region (cKO region), homologous arms and cKO region will be generated by PCR using BAC clone RP23-113O21 as template. The *gRNA* to *Pvt1* gene (*gRNA-A1*: TTGCATACCACAGATAATAGTGG; *gRNA-A2*: TCACCATACCAGGCACATATAGG), the donor vector containing loxP sites and *Cas9* mRNA were co-injected into fertilized mouse eggs to generate *Pvt1* conditional knockout offspring. For *Trim56* conditional knockout mouse, exon 1-3 selected as cKO region. To engineer the targeting vector, homologous arms and cKO region will be generated by PCR using BAC clone RP24-359E6 as template. The *gRNA* to *Trim56* gene (*gRNA-A1*: CCTGCTTTTCCGCTGGCAAGAGG; *gRNA-A2*: CAGCTAAAGCGGCTTTTGGCTGG), the donor vector containing loxP sites and *Cas9* mRNA were co-injected into fertilized mouse eggs to generate *Trim56* conditional knockout offspring. F0 founder animals were identified by PCR followed by sequence analysis, which were bred to wild-type mice to test germline transmission and F1 animal generation. Inter-cross heterozygous targeted mice to generate homozygous targeted mice, then breed a homozygous targeted mouse with a *NPHS2*-Cre delete mouse to generate podocyte-specific *Pvt1* and/or *Trim56* knockout mice (*Cre*^*+*^*/Pvt1*^*flox/flox*^*; Cre*^*+*^*/Trim56*^*flox/flox*^), mice with two WT alleles and *Cre* expression were used as controls (*Cre*^*+*^*/Pvt1*^+/+^*; Cre*^*+*^*/Trim56*^+/+^).

### Animal model

All experiments were performed under the approval of the Guide for the Care and Use of Laboratory Animals and were endorsed by the Animal Ethics Committee of Shandong Provincial Hospital Affiliated to Shandong First Medical University (NSFC: NO.2020-901). The DKD model was induced in male C57BL/6 mice (8 weeks of age) with intraperitoneal administration of streptozotocin (STZ) (Sigma, dissolved in 0.1 mM citrate buffer, pH = 4.5) at 50 mg/kg each day for 5 consecutive days. Citrate buffer-injected mice functioned as the control groups, DKD model was established successfully until the level of blood glucose ≥ 16.7 mmol/L.

Blood glucose, systolic blood press (SBP), body weight and kidney weight were examined, urine was collected via metabolic cages for 24 h at 16 weeks (6 weeks after DKD initiation), at 18 weeks (8 weeks after DKD initiation), at 22 weeks (12 weeks after DKD initiation), at 26 weeks (16 weeks after DKD initiation), and at 30 weeks of age (20 weeks after DKD initiation) before the mice were sacrificed. Albuminuria was determined by the urine albumin-creatinine ratio (ACR). The kidney weight was measured before sacrificing the mice at 30 weeks of age (20 weeks after DKD initiation).

### Cell culture and treatment

Conditionally immortalized human podocyte cell line was a kind gift from Professor Peter Mundel, cells were cultured as previously described [13]. To explore the function of AMPKα, podocytes were exposed to aminoimidazole carboxamide ribonucleotide (AICAR) (2 mmol/L) (MCE, USA) or compound C (5 μmmol/L) (MCE, USA) for 4 h before collection or as indicated.

For primary podocytes, the separation of glomeruli from mouse renal cortices was performed through the filters with 250, 100, and 70 mm pores step by step before incubation in type I collagen-coated plates in RPMI 1640 medium. The isolated glomeruli were removed 7 days later, and the primary podocytes were filtered through a filter with 40 mm pores after trypsinization.

### Plasmids, small-interfering RNA, and transfection

The Myc-AMPKα (WT, K60R, K379R, K470R), Flag-TRIM56 (full length-FL, ∆RING, ∆RING + B-box, ∆N, ∆C), and HA-Ubiquitin, were synthesized by BioSune (China). Ubiquitin mutants K48R and K63R (the lysine at positions 48 and 63 substituted by arginine) were generated from the wild-type ubiquitin expression vector by point mutation. The *PVT1* overexpression plasmid (oe-*PVT1*), *TRIM56* overexpression plasmid (oe-*TRIM56*), the small-interfering RNA targeting *TRIM56*, control (si-NC), and antisense oligonucleotides targeting *PVT1 (PVT1*-ASO) were synthesized by Genomeditech (Shanghai, China). The plasmids and siRNA (3 μg) were used to transfect cells with the aid of Lipofectamine2000 (Invitrogen, USA). The siRNA sequences used were as follows: *PVT1*-ASO: 5’-CTTTTAGTATCCTGAAATGTG-3’; si*-ALKBH5*: 5’*-*GCUGCAAGUUCCAGUUCAAtt-3’ and 5’-UUGAACUGGAACUUGCAGCtt-3’; si*-TRIM56*: 5’*-*GUACUUGGUGGUGUCACUUAGTT-3’ and 5’-CUAAGUGACACCACCAAGUACTT-3’; si-NC: 5’-UUCUCCGAACGUGUCACGUdTdT-3’ and 5’-ACGUGACACGUUCGGAGAAdTdT-3’.

### RNA fluorescence in situ hybridization (RNA-FISH)

The in situ detection of *PVT1* was performed using Ribo^TM^ Fluorescent In Situ Hybridization Kit (RiboBio, China) in accordance with manufacturer’s instructions. The fluorescent images were viewed and captured under confocal laser scanning microscope (Leica, Germany), followed by the nucleus were stained using 4,6-diamidino-2-phen-ylindole (DAPI) working solution.

### Quantitative RT-PCR analysis (qRT-PCR)

Plasma RNA was extracted using a BIOG cfRNA Easy Kit (BIOG, Changzhou, China) according to the manufacturer’s instructions. Total RNA was extracted from cells and kidney tissues using TRIzol Reagent (Takara, China) as we described previously. RNA samples were reverse transcribed and amplified. β-actin was used as internal controls, and the relative expression of different genes was calculated following the 2^–∆∆CT^ method or following a log transformation after the 2^–∆CT^ method. For mtDNA detection, DNA was isolated from whole-cell extracts and cytosolic fractions [14] using the MiniBEST Universal Genomic DNA Extraction Kit (Takara, China). mtDNA and nuclear DNA (nDNA) were determined by amplifying a short region of the mitochondrially encoded tRNA-LeuUUR gene and β2-microglobulin [15], and mtDNA copy number was calculated as the mtDNA/nDNA ratio. The primers were synthesized by BioSune (China) and the sequences were described in Table S1.

### Western blotting, immunoprecipitation and ubiquitination assay

The detailed protocol was elaborated previously.^(13)^ For ubiquitination assay, cells were co-transfected with indicated plasmids for 24 h. Subsequently, cells were incubated with MG132 (20 μM) for 4 h to prevent proteasome degradation. Cell lysates were immunoprecipitated and analyzed using immunoblotting with indicated antibodies. The antibodies are listed as follows: UQCRC2, SDHB, NDUFB8, MTCO1, ATP5A, ALKBH5, TRIM56,TFAM, p65, p-p65, DRP1, BAX, AMPKα1/α2 (Abcam, USA), p-DRP1 (Ser616) (Cell signaling, USA), AMPKα, PGC-1α, Myc-tag, Flag-tag, HRP-conjugated goat anti-rabbit/mouse IgG, and β-actin (Proteintech, USA).

### Mitochondrial membrane potential (ΔΨm) assay

The mitochondrial ΔΨm was measured with a cell-permeant dye tetramethylrhodamine, methyl ester (TMRM) in concert with the manufacturer’s instructions (Invitrogen, USA). At first, TMRM staining solution was prepared freshly by adding 10 μL of the 100 μM stock solution to 10 mL of RPMI-1640 medium. Cells in a 6-well plate were cultured in an incubator of 5% CO_2_ at 37 °C, after removing the cell growth medium, the freshly prepared TMRM staining solution was added to plate and then cells were incubated for 30 min at 37 °C. Next, cells were washed twice with warm PBS. Finally, images were observed and scanned with inverted fluorescence microscope (Leica Microsystems GmbH, Germany) at 488 nm excitation and 570 ± 10 nm emission filter for detection or compatible settings.

### Measurement of mtROS

The mtROS was detected by MitoSOX^TM^ Red mitochondrial superoxide indicator for live-cell imaging (Invitrogen, USA). Firstly, dissolve mitochondrial superoxide indicator (50 μg) in 13 μL dimethylsulfoxide (DMSO) to get MitoSOX^TM^ reagent stock solution (5 mM), and then the stock solution was diluted further in HBSS buffer to make a working solution (5 μM). Next, 2 mL working solution was applied to cover podocytes adhering to coverslips in 6-well plate. Incubate cells for 20 min at 37 °C in dark conditions, followed by washing cells gently with warm buffer. In the end, the stained cells with counterstains as desired and mount in warm buffer for imaging, inverted fluorescence microscope (Leica Microsystems GmbH, Germany) was utilized to view and capture images.

### RNA immunoprecipitation (RIP) assays

RIP assay was performed by Magna RIP^TM^RNA-Binding Protein Immunoprecipitation Kit (Millipore, USA) in conformity with the detailed protocol, respectively. The enrichment of co-precipitated RNAs were eluted and purified and then detected by qRT-PCR.

### RNA pull-down assay

RNA pull-down assay was employed using Pierce™ Magnetic RNA-Protein Pull-Down Kit (Thermo Scientific, USA). Human *PVT1* cDNAs (sense and antisense; RiboBio Technology) and the truncated constructs were transcribed using TranscriptAid T7 High Yield Transcription Kit (Thermo Scientific, USA). Then the RNA 3’ End Desthiobiotinylation Kit (Thermo Scientific, USA) was applied to label transcripts with biotin in 3’ end to generate RNA probes. And RNA pull-downs were performed using Magnetic RNA-Protein Pull-Down Kit (Thermo Scientific, USA) in accordance with the manufacturer’s instructions. The eluted proteins were determined by western blotting or mass spectrometry.

### Immunofluorescence

Paraformaldehyde-fixed cells or kidney sections were stained with primary antibodies at 4 °C overnight, and then incubated by fluorescent-dye conjugated secondary antibody (Dylight 594-or 488) the next day at 37 °C. All operations were performed in subdued lighting, out of direct sunlight or direct bright fluorescence lighting. Inverted fluorescence microscope (Leica Microsystems GmbH, Germany) was employed to capture pictures. The following antibodies were used: podocin, WT-1, p65, goat anti-rabbit IgG H&L (Alexa Fluor 594 or 488), goat anti-mouse IgG H&L (Alexa Fluor 488) (Abcam, USA), dsDNA (Cayman Chemical, USA), and Tom20 (Proteintech, USA). The number of WT-1 was measured in 5 glomeruli per mouse, and ImageJ 10.2 software was used to measure the intensity of immunostaining in the glomeruli.

### In Situ Proximity ligation assay (PLA)

PLA was performed by Duolink In Situ PLA Kit (Sigma, USA) in conformity with the detailed protocol. Briefly, cells cultured on coverslips were fixed, permeabilized, and then incubated with indicated antibodies (anti-TRIM56, anti-AMPKα) at 4 °C overnight, followed by incubation with Duolink PLA anti-Mouse PLUS and PLA anti- Rabbit PLUS probes at 37 °C for 1 h. After ligation and the amplification at 37 °C for 100 min, cells were stained with DAPI and captured by Inverted fluorescence microscope (Leica Microsystems GmbH, Germany).

### Histology staining

Kidney sections were stained with Periodic Acid-Schiff (PAS) according to the minute protocols. The relative mesangial areas were determined as previously described [13].

### Transmission electron microscopy (TEM)

Fresh renal tissues (< 1 cm^3^) or cell pellet were fixed in TEM stationary solution at 4 °C overnight, and then fixed in 1% osmium tetroxide at 4 °C for 2 h, followed by the specimens were dehydrated plying alcohol and embedded in Epon 812 (90529-77-4, SPI). After polymerization at 60 °C for 48 h, ultra-thin sections were stained with uranyl acetate and lead citrate, and finally observed under TEM (Hitachi, Tokyo, Japan). Foot process (FP) widths of glomeruli and thickness of the GBM was measured as previously described [13].

### Seahorse XF Cell Mito Stress Test

Turn on the Agilent Seahorse XFe/XF Analyzer, and let it warm up overnight. Plate podocytes at a number of 2 × 10^4^ in the Seahorse XF Cell Culture Microplate using the RPMI 1640 medium. Then hydrate a sensor cartridge in Seahorse XF Calibrant at 37 °C in a non-CO_2_ incubator overnight. The next day, using 1 mM pyruvate, 2 mM glutamine, and 10 mM glucose as a starting point to prepare assay medium by supplementing Seahorse XF Base Medium and adjusted to pH =7.4, subsequently, the assay medium was warmed to 37 °C. Then the Seahorse XF Cell Mito Stress Test Kit containing oligomycin, FCCP and Rotenone/antimycin A was employed according to the manufacturer’s instructions. And the compound was prepared for loading into the appropriate ports of a hydrated sensor cartridge. Finally, the Seahorse XF Mito Stress Test Report Generator was used to calculate the Seahorse XF Cell Mito Stress Test parameters automatically.

### Enzyme linked immunosorbent assay (ELISA)

Albumin in urine was detected by Mouse ALB (Albumin) ELISA Kit (Elabscience, China), creatinine in urine was detected by Creatinine (Cr) Colorimetric Assay Kit (Elabscience, China). TNF-α in human plasma was quantified using Human TNF-α ELISA Kit (Elabscience, China). ELISA was performed according to the manufacturer’s protocol. Absorbances were read by Multitask Microplate Reader (Thermo Fisher Scientific, USA).

### Statistical analysis

Statistical significance was calculated with data from not less than three independent experiments, GraphPad Prism 6 was applied for data analysis exerting Student t-test for two groups and one-way ANOVA for more than two groups as well. Data are described as the mean ± S.D. For all statistical tests, significance was considered as *P* < 0.05.

## Results

### DKD is associated with increased expression of *PVT1* and mitochondria damage

**Fig. 1:**
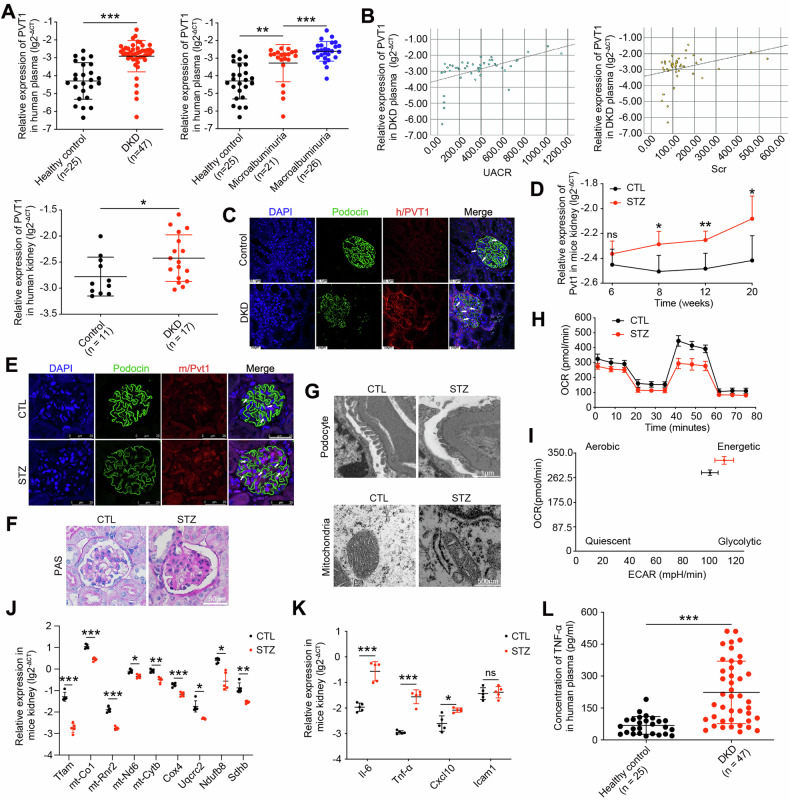
DKD is associated with increased expression of *PVT1* and mitochondria damage. **A** Levels of *PVT1* in the plasma and kidney tissues from controls and patients with DKD. **B** The correlation between *PVT1* levels and UACR (*r* = 0.535, *P* < 0.01) or Scr (*r* = 0.318, *P* = 0.029). **C** RNA-FISH combined with immunofluorescence illustrated the distribution of *PVT1* and its relative position to podocin by confocal laser scanning microscope (*n* = 3). The podocytes were labeled with arrows. **D** Levels of *Pvt1* in the kidney tissues from CTL mice and STZ mice at 6 weeks (*n* = 7), 8 weeks (*n* = 7), 12 weeks (*n* = 7), and 20 weeks (*n* = 5) after the onset of diabetes. **E** RNA-FISH combined with immunofluorescence illustrated the distribution of *Pvt1* and its relative position to podocin in paraffin-embedded mouse sections by confocal laser scanning microscope (*n* = 5). The podocytes were labeled with arrows. **F** PAS staining of glomeruli from CTL mice and STZ mice at 20 weeks after DKD initiation (*n* = 5). **G** TEM image of podocytes, GBM, and mitochondria in primary podocytes from CTL mice and STZ mice at 20 weeks after DKD initiation (*n* = 5). **H** Measurement of the mitochondrial OCR of primary podocytes from CTL mice and STZ mice at 20 weeks after DKD initiation (*n* = 5). **I** Energy phenotype profile (EPP) was mentioned. **J** Relative mRNA levels of *Tfam* and mitochondrial OXPHOS genes in CTL mice and STZ mice at 20 weeks after DKD initiation (*n* = 5). **K** Relative mRNA levels of proinflammatory cytokines (*Il-6, Tnf-α, Cxcl10*, and *Icam1*) in CTL mice and STZ mice at 20 weeks after DKD initiation (*n* = 5). **L** ELISA showed the levels of TNF-α in the plasma from healthy subjects (*n* = 25) and clinical patients with DKD (*n* = 47). Error bars represent the mean± S.D, *P < 0.05, **P < 0.01, and ***P < 0.001.

To elucidate the role of *PVT1* in DKD patients, qRT-PCR was applied to quantify *PVT1* levels in plasma of subjects with or without DKD, and we found an upregulated *PVT1* expression in patients with DKD compared to controls (Fig. 1A). Compared to the control, *PVT1* expression was increased in patients with microalbuminuria (30-300 mg/g) and macroalbuminuria (> 300 mg/g) (Fig. 1A), and the levels of *PVT1* expression correlated positively with UACR (r = 0.535, *P* < 0.01) and serum creatinine (Scr) (*r* = 0.318, *P* = 0.029) (Fig. 1B). Concurrently, we observed significantly increased *PVT1* expression in the kidney tissues of patients with DKD compared to those controls (Fig. 1A). RNA-FISH combined with immunofluorescence demonstrated that *PVT1* was localized predominantly in podocytes as it co-localized with podocin and its staining intensity in podocytes was significantly increased in DKD patients (Fig. 1C). The important clinical characteristics of the subjects are listed in Table S2.

Since *PVT1* exhibits robust evolutionary conservation between humans and mice, we liked to study its expression in mouse models of DKD. We induced diabetes in mice with streptozotocin (STZ) (Fig. S1A). As shown in Fig. 1D, while no significant difference in *Pvt1* expression was observed between the control mice (CTL) and STZ mice at 6 weeks after induction of diabetes, a significant increase in *Pvt1* expression occurred after 8 weeks, and this increase became more apparent at 12 weeks as diabetes progressed. Also, RNA-FISH combined with immunofluorescence reveled that *Pvt1* was found in both the nucleus and cytoplasm and that its expression increased in the podocytes of STZ mice when compared with CTL mice at 20 weeks after induction of diabetes (Fig. 1E). Periodic acid-Schiff (PAS) taining demonstrated significant matrix expansion in mesangial area and glomerular hypertrophy in STZ mice (Fig. 1F). Transmission electron microscopy (TEM) analysis revealed reduced, retracted and fused podocyte foot processes, accompanied by GBM thickening in STZ mice glomeruli compared with CTL group (Fig. 1G). The relative mesangial areas (%), glomerular volume, foot process width and GBM thickness was quantified (Fig. S1B). TEM also showed mitochondrial swelling, mitochondria fragmentation and vacuolization in podocytes of STZ mice but not in control mice (Fig. 1G). The results of OCR revealed that compared with primarily cultured podocytes from the control groups, the STZ mice had decreased basal respiration, maximal respiration and spare respiratory capacity, accompanied by decreased ATP production (Figs. 1H and S1C), thereby switching to quiescent metabolism which was evidenced by energy phenotype profile (EPP) (Fig. 1I). Furthermore, we found the mRNA levels of mitochondrial OXPHOS genes (*mt-Co1, mt-Rnr2, mt-Nd6, mt-Cytb, Cox4, Uqcrc2, Ndufb8, and Sdhb*) and *Tfam* were significantly lower in STZ mice as compared to controls (Fig. 1J).

Studies have reported that mitochondrial dysfunction could activate inflammatory responses [16, 17], thus we detected the mRNA levels of inflammatory cytokines. We observed that *Il-6, Tnf-α* and *Cxcl10* were increased in isolated kidney glomeruli from DKD mice, while there was no significant change of *Icam1* between the two groups (Fig. 1K). We also found that the concentration of TNF-α was increased in plasma of patients with DKD compared to those of controls (Fig. 1L). Collectively, we concluded that human and mouse with DKD is characterized by increased expression of *PVT1*, and mitochondria damage and increased inflammatory response.

### *PVT1* deletion ameliorated podocytes injury in STZ-induced diabetic mice

**Fig. 2:**
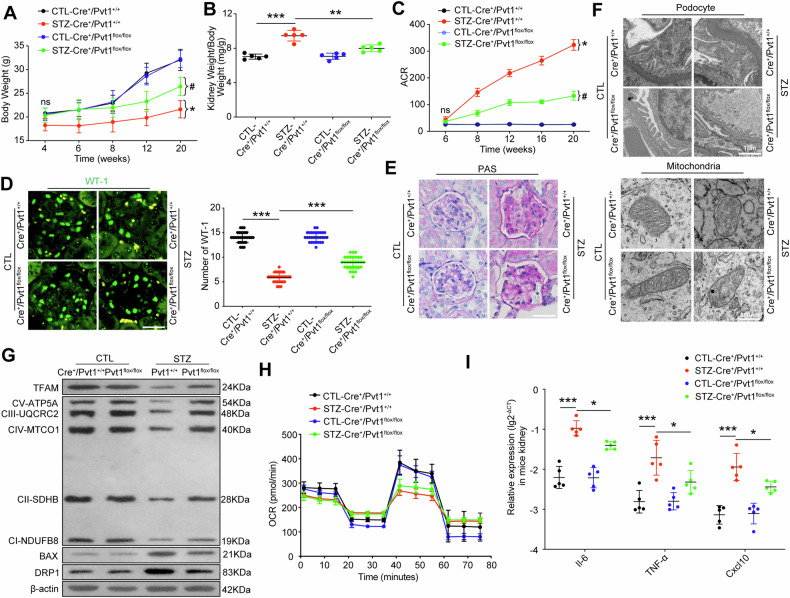
*PVT1* deletion ameliorated podocyte injury in STZ-induced diabetic mice. **A** Temporal changes in body weights were measured respectively at 4 weeks (*n* = 6), 6 weeks (*n* = 6), 8 weeks (*n* = 5), 12 weeks (*n* = 5), and 20 weeks (*n* = 5) after DKD initiation. *P < 0.05 vs CTL-*Cre*^*+*^*/Pvt1*^*+/+*^, ^#^*P* < 0.05 vs STZ-*Cre*^*+*^*/Pvt1*^*+/+*^. **B** Kidney weight to body weight ratio was elevated in mice at 20 weeks after DKD initiation (*n* = 5). **C** Temporal urinary albumin-to-creatinine ratio (ACR) of mice were detected respectively at 6 weeks (*n* = 6), 8 weeks (*n* = 5), 12 weeks (*n* = 5), 16 weeks (*n* = 5), and 20 weeks (*n* = 5). *P < 0.05 vs CTL-*Cre*^*+*^*/Pvt1*^*+/+*^, ^#^*P* < 0.05 vs STZ-*Cre*^*+*^*/Pvt1*^*+/+*^. **D** Representative fluorescence images of WT-1 in glomeruli isolated from the four groups at 20 weeks after DKD initiation (*n* = 5). Scale bar = 50 μm. **E** PAS staining of glomeruli from the four groups at 20 weeks after DKD initiation (*n* = 5). Scale bar = 50 μm. **F** TEM image of podocytes and, GBM, and mitochondria in primary podocytes from the four groups at 20 weeks after DKD initiation (*n* = 5). **G** Expression of TFAM, BAX, DRP1, and mitochondrial OXPHOS proteins in primary podocytes from the four groups at 20 weeks after DKD initiation (*n* = 3). **H** Measurement of the mitochondrial OCR of primary podocytes from mice at 20 weeks after DKD initiation (*n* = 5). **I** Relative mRNA levels of *Il-6, Tnf-α*, and *Cxcl10* in mice at 20 weeks after DKD initiation (*n* = 5). Error bars represent the mean ± S.D, *P < 0.05, **P < 0.01, and ***P < 0.001.

To explore whether *PVT1* upregulation is associated with mitochondria damage and increased inflammatory response in DKD, we deleted *Pvt1* in kidney podocytes by crossbreeding *Nphs2-Cre* and Pvt1^*flox/flox*^ mice to generate *Nphs2-Cre/Pvt1*^*flox/flox*^ mice (*Cre*^*+*^*/Pvt1*^*flox/flox*^ mice) (Figure S2A), which was identified by tail genotyping and RNA-FISH (Fig. S2B and S2C). After induce of DKD mouse model using STZ (Fig. S2D), we found that the blood glucose levels were significantly higher in the STZ-treated groups than in the control mice at every observation point, while there were no significant differences in blood glucose levels between the STZ-*Cre*^*+*^*/Pvt1*^*+/+*^ and STZ-*Cre*^*+*^*/Pvt1*^*flox/flox*^ groups (Fig. S2E), and there were no significant differences in SBP among the four groups before 20 weeks (Fig. S2F). Compared to the CTL-*Cre*^*+*^*/Pvt1*^*+/+*^ mice, STZ-treated *Cre*^*+*^*/Pvt1*^*+/+*^ (STZ-*Cre*^*+*^*/Pvt1*^*+/+*^) mice showed decreased body weight at 6 weeks, 8 weeks, 12 weeks and 20 weeks after DKD initiation, while the STZ-*Cre*^*+*^*/Pvt1*^*flox/flox*^ mice showed less reduction of body weight at 20 weeks after DKD initiation compared to STZ-*Cre*^*+*^*/Pvt1*^*+/+*^ mice (Fig. 2A). Moreover, at 20 weeks after DKD initiation, the kidney weight to body weight ratio in the STZ-*Cre*^*+*^*/Pvt1*^*+/+*^ mice was higher than that in the control group, whille the ratio in the STZ-*Cre*^*+*^*/Pvt1*^*flox/flox*^ mice was decreased compared with that in the STZ-*Cre*^*+*^*/Pvt1*^*+/+*^ group (Fig. 2B), suggesting kidney hypertrophy is prevented in the *Pvt1* knockout mice. STZ-treated mice exhibited a significantly higher ACR levels compared with the control mice at 8 weeks, 12 weeks, 16 weeks and 20 weeks after DKD initiation, while the STZ-*Cre*^*+*^*/Pvt1*^*flox/flox*^ mice exhibited lower albuminuria levels than those of the STZ-*Cre*^*+*^*/Pvt1*^*+/+*^ mice after 8 weeks of DKD (Fig. 2C). Additionally, we found that compared with the control groups, the STZ-*Cre*^*+*^*/Pvt1*^*+/+*^ mice exhibited decreased staining for WT-1, whilst knockout of *Pvt1* restored expression of this podocyte marker (Fig. 2D). Besides, PAS staining showed apparent mesangial expansion and glomerular hypertrophy, which were ameliorated in *Pvt1* knockout mice (Fig. 2E). Notably, TEM showed the morphological changes of podocytes in the glomerular area of the DKD mice, while these lesions induced by STZ were substantially ameliorated in *Pvt1* knockout mice (Fig. 2F). The relative mesangial areas (%), glomerular volume, foot process width and GBM thickness were quantified as shown in Fig. S2G.

Concomitantly, TEM showed the morphological changes of mitochondria in primarily cultured podocytes, which were consistent with the results as we described above, while *Pvt1* deficiency ameliorated these mitochondrial lesions in diabetic mice (Fig. 2F). We also examined the protein levels of TFAM, BAX, DRP1 and OXPHOS-related proteins in primary podocytes from the four groups of mice at 20 weeks after DKD initiation by western blotting analysis and confirmed that knockout of Pvt1 improved mitochondrial function in diabetic mice (Fig. 2G). Next, we detected the changes of mitochondrial respiratory function in primary podocytes and found that compared with the CTL-*Cre*^*+*^*/Pvt1*^*+/+*^ mice, the STZ-*Cre*^*+*^*/Pvt1*^*+/+*^ mice were decreased at basal respiration, maximal respiration and spare respiratory capacity, and accompanied by decreased ATP production, knockout of *Pvt1* clearly restored maximal respiration and ATP production in podocytes, whereas it had no significant effects on basal respiration and spare respiratory capacity (Figs. 2H and S2H), indicating that although *Pvt1* depletion could improve the mitochondrial function of podocytes, it does not enhance the adaptability or flexibility of podocytes. Also, EPP revealed that the primary podocytes from STZ-*Cre*^*+*^*/Pvt1*^*+/+*^ mice switched to glycolytic metabolism as compared to normal controls, while the EPP of primary podocytes changed back to energetic metabolism at least partially in *Pvt1* knockout mice (Fig. S2I). Moreover, increased transcript levels of proinflammatory cytokine genes (*Il-6, Tnf-α and Cxcl10*) in primary podocytes of diabetic mice were markedly reversed by *Pvt1* deletion (Fig. 2I).

### Silence of *PVT1* alleviated mitochondrial dysfunction and inflammation in high glucose-stimulated podocytes

**Fig. 3:**
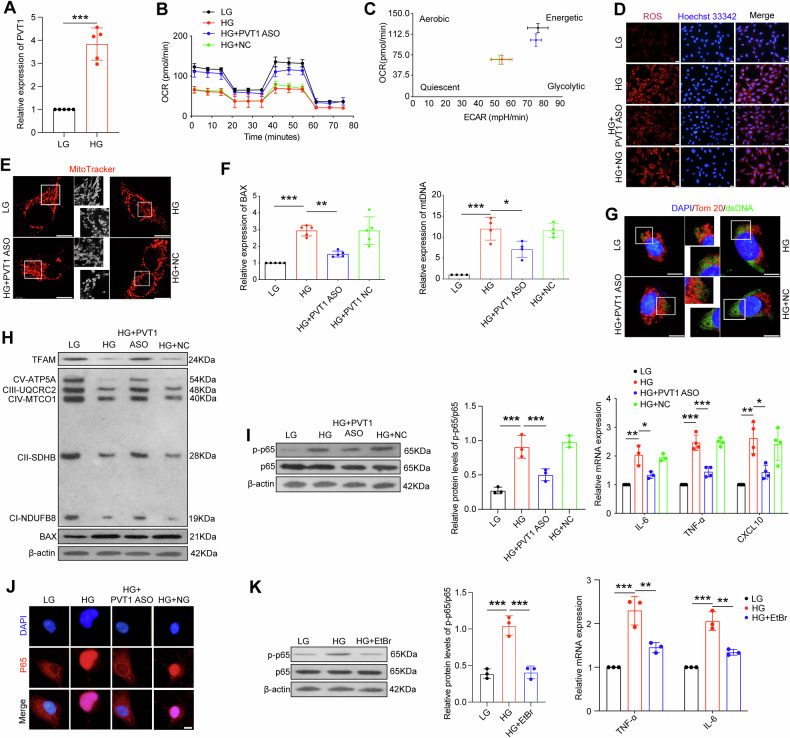
Silence of *PVT1* alleviated mitochondrial dysfunction and inflammation in high glucose-stimulated podocytes. **A** The RNA levels of PVT1 in podocytes under different conditions (*n* = 5). **B** Measurement of the mitochondrial OCR of podocytes under different conditions (*n* = 5). **C** Energy phenotype profile (EPP) was mentioned. **D** Production of mtROS in podocytes (*n* = 5). Scale bar = 50 μm. **E** Representative fluorescence images of MitoTracker in podocytes cultured under the indicated conditions (*n* = 4). Scale bar =50 μm. **F** qRT-PCR measurement of the mRNA levels of *BAX* in podocytes (*n* = 5) and levels of *mtDNA* in cytoplasm of podocytes (*n* = 4). **G** Representative fluorescence images of podocytes with anti-Tom 20 (red) and anti-dsDNA (green) antibodies (*n* = 4). Scale bar = 50 μm. **H** Expression of TFAM, BAX, and mitochondrial OXPHOS proteins in podocytes under different conditions by western blotting (*n* = 4). **I** Expression of p-p65, p65, *IL-6*, *TNF-α*, and *CXCL10* in podocytes under different conditions. **J** Representative fluorescence images of podocytes with anti-p65 antibody (*n* = 3). Scale bar = 50 μm. **K** Expression of p-p65, p65, *IL-6*, and *TNF-α* in podocytes in the presence of EtBr (1.0 μg/ml) to block the replication of mtDNA (*n* = 4). Error bars represent the mean± S.D, *P < 0.05, **P < 0.01, and ***P < 0.001.

Firstly, we detected the level of PVT1 expression in high glucose-stimulated podocytes, and the results demonstrated that high glucose treatment resulted in an increase of PVT1 expression (Fig. 3A). To further determine the functions of *PVT1* in podocytes under hyperglycemia conditions, we constructed *PVT1* ASO lentivirus (Fig. S3A). The results of OCR revealed that the mitochondrial respiration of hyperglycemia-treated podocytes was decreased at both basal and maximal levels, and accompanied by decreased spare respiratory capacity and ATP production, thereby switching to quiescent metabolism, while the transfection of *PVT1* ASO ameliorated mitochondrial damage status (Figs. 3B, C, S3B). We also found a pronounced increase of fluorescence intensity of mtROS in hyperglycemia-treated podocytes, in contrast, *PVT1* suppression prevented HG-induced mtROS accumulation (Fig. 3D). Given that mitochondrial function was mainly associated with mitochondrial morphology, we used mitoTracker, a cell-permeant dye, to observe the morphology change of mitochondria in different groups. The long filamentous mitochondria with a thread-like appearance was observed in LG group, while podocytes treated with HG showing shortened punctate mitochondria, in contrast, the disrupted mitochondrial morphology and higher mitochondrial fission were mitigated via silencing of *PVT1* (Fig. 3E). Moreover, mitochondrial membrane permeability, estimated by mRNA expression of *BAX*, which is a component of the permeability transition pore, was increased in hyperglycemia environment, and this effect was accompanied by the upregulation of mtDNA in the mitochondria-free cytosol. However, the use of *PVT1* ASO decreased the expression of *BAX* and the content of cytosolic mtDNA (Fig. 3F). Next, to visualize the release of mtDNA from damaged mitochondria, we applied dual immunostaining of the dsDNA and Tom20, a mitochondria outer membrane protein, which revealed that the most of dsDNA were contained within mitochondria and were surrounded by a continuous mitochondria outer membrane in LG, while HG treatment significantly increased the cytosolic dsDNA signals, suggesting that the dsDNA, including mtDNA, were released into the cytosol by damaged mitochondria following the stimulation of hyperglycemia in podocytes, yet the suppression of *PVT1* mitigated the integrity of mitochondrial outer membrane and curtailed the cytosolic dsDNA signals (Fig. 3G). We also found that *PVT1* ASO lowered the expression of BAX, and increased the expression of TFAM and OXPHOS-related proteins (CV-ATP5A, CIII-UQCRC2, CIV-MTCO1, CII-SDHB, and CI-NDUFB8) as compared to HG group (Fig. 3H).

Besides, we observed that silence of *PVT1* in podocytes inhibited the expression of NF-κB p-p65, and alleviated the increase of proinflammatory cytokines *IL-6*, *TNF-α* and *CXCL10* in HG-treated podocytes(Fig. 3I), consistently with the results of Immunofluorescence, showing that inhibition of *PVT1* alleviated hyperglycemia-induced nuclear translocation of the p65 subunit of NF-κB (Fig. 3J). To further investigate whether the presence of mtDNA in podocytes can affect inflammatory cytokine activation, we used ethidium bromide (EtBr) to block the replication of mtDNA but not nuclear DNA at 1.0 μg/ml, and found that EtBr abolished the HG-induced phosphorylation NF-κB p65 and increase of proinflammatory cytokines (Fig. 3K). Taken together, these findings strongly implicated that inhibition of *PVT1* alleviated mitochondrial dysfunction and inflammation in podocytes provoked by hyperglycemia in vitro.

### *PVT1* participated in high glucose-induced mitochondrial dysfunction and inflammation through promoting TRIM56 expression

**Fig. 4:**
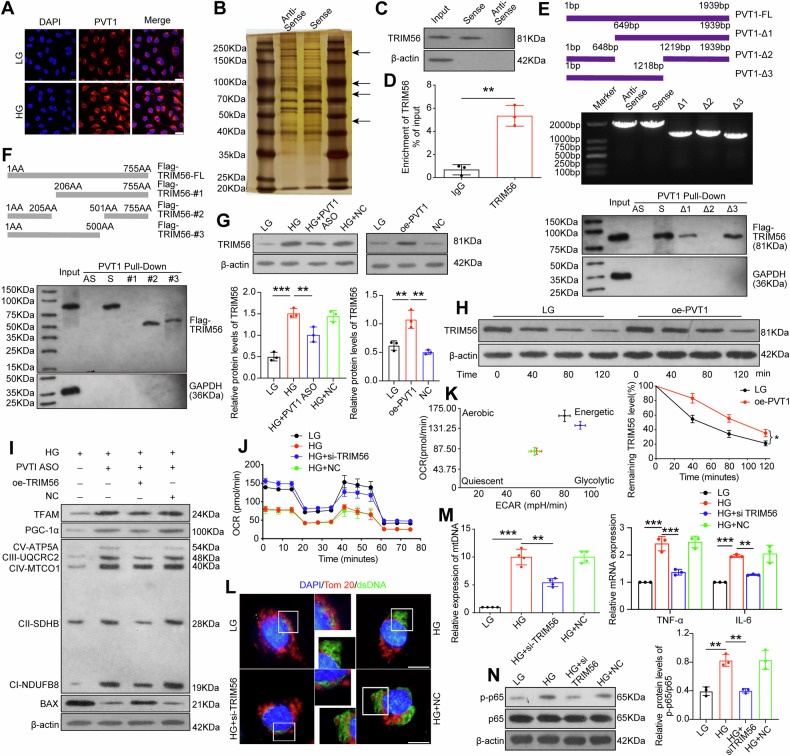
*PVT1* participated in high glucose-induced mitochondrial dysfunction and inflammation through promoting TRIM56 expression. **A** RNA-FISH revealed the location and expression of *PVT1* under LG or HG conditions in podocytes (*n* = 4). Scale bar = 25 μm. **B** Silver staining of RNA pull-down in podocytes. The differentially expressed proteins have been indicated by arrows. **C** The eluted proteins from RNA pull-down assay were detected by SDS-PAGE by using anti-TRIM56 antibody (*n* = 3). **D** RIP assay using TRIM56 antibody was carried out in podocytes (*n* = 3). **E** *PVT1* truncations (FL, ∆1, ∆2, and ∆3) were constructed, identified by PCR, and RNA pull-down assays were performd to map the binding region of *PVT1* to TRIM56 in podocytes after transfection of Flag-TRIM56. **F** TRIM56 truncations (FL, #1, #2, and #3) were constructed and RNA pull-down assays were performd to map the binding region of TRIM56 to *PVT1* in podocytes after transfection of indicated vectors. **G** Western blotting detected the expression of TRIM56 in podocytes under different conditions. **H** Western blotting detected protein levels of TRIM56 in podocytes after treatment with CHX (20 mg/ml) at the indicated time points and relative TRIM56 levels were quantified. **I** Expression of TFAM, PGC-1α, BAX, and mitochondrial OXPHOS proteins in podocytes under different conditions (*n* = 3). **J** Measurement of the mitochondrial OCR of podocytes (*n* = 3). **K** Energy phenotype profile (EPP) was exhibited. **L** Representative fluorescence images of podocytes with anti-Tom 20 (red) and anti-dsDNA (green) antibodies (*n* = 3). Scale bar = 50 μm. **M** Relative expression of *mtDNA* in cytoplasm of podocytes cultured under the indicated conditions (*n* = 4). **N** Expression of p-p65, p65, *TNF-α*, and *IL-6* in podocytes under different conditions. Error bars represent the mean ± S.D, *P < 0.05, **P < 0.01, and ***P < 0.001.

To clarify the molecular mechanism of PVT1 in HG-induced mitochondrial dysfunction and inflammation, we evaluated its subcellular localization and RNA-FISH revealed that *PVT1* was located both in the nucleus and the cytoplasm of podocyte, and that the intensity of *PVT1* staining was evidently increased after HG treatment (Fig. 4A), which consistent with our previous data, indicating that PVT1 may function by interacting with RNA-binding proteins (RBPs). By performing RNA pull-down assay and searched MitoCarta3.0 datasets (https://www.broadinstitute.org/mitocarta/mitocarta30-inventory-mammalian-mitochondrial-proteins-and-pathways), we selected tripartite motif (TRIM) 56, an E3 ubiquitin ligase, as a protein partner of *PVT1*(Fig. 4B). Western blot analysis validated that TRIM56 was notably enriched by *PVT1* in podocytes (Fig. 4C). Also, RIP assay demonstrated a physical association between *PVT1* and TRIM56 in podocytes (Fig. 4D). Then, we searched the fragment of *PVT1* that was responsible for the interaction with TRIM56 by deletion-mapping system. We constructed three *PVT1* truncations: i) ∆1 (649-1939 bp); ii) ∆2 (1-648 bp, 1219-1939 bp) and iii) Δ3(1-1218 bp) and found that *PVT1* ∆1 and Δ3 truncations interacted with Flag-TRIM56 (Fig. 4E), suggesting that the common sequence of *PVT1* ∆1 and Δ3 (649-1218 bp) is required for association with Flag-TRIM56. Meanwhile, we constructed three TRIM56 truncations: i) #1 (206-755 aa); ii) #2 (1-205 aa, 501-755 aa) and iii) #3(1-500 aa), RNA pull-down revealed that Flag-TRIM56 FL (1-755 aa), Flag-TRIM56 #2 and #3 truncations interacted with *PVT1*(Fig. 4F), suggesting that the common region of TRIM56 #2 and #3 (1-205 aa), which contain N-terminal RING-finger domain (21-59 aa) and B-box domain (164-205 aa) of TRIM56, is indispensable for the interaction with *PVT1* in podocytes. Subsequently, we detected the levels of TRIM56 in kidney biopsies between DKD patients and healthy controls by immunohistochemical staining, and observed more intense staining of TRIM56 in the glomeruli of DKD patients when compared with healthy controls. Correlation analyses showed that the glomerular TRIM56 expression positively correlated with UACR (r = 0.5552, *P* = 0.011), and negatively correlated with eGFR (*r* = −0.5064, *P* = 0.0227) (Figure S4A, B). We also found that both HG treatment and overexpression of *PVT1* markedly increased TRIM56 levels, while silencing *PVT1* significantly suppressed the expression of TRIM56 in cultured podocytes (Fig. 4G), indicating that *PVT1* acts as an upstream regulator of TRIM56. Immunoblotting analysis showed that the half-life of TRIM56 was substantially prolonged following *PVT1* overexpression, as determined by cycloheximide (CHX) at the indicated time points (Fig. 4H), suggesting that *PVT1* likelys regulates TRIM56 expression at the post-translational level.

Next, we explored whether *PVT1* paticipated in mitochondrial dysfunction and inflammation in podocytes via downstream TRIM56, and we constructed *TRIM56* siRNA and *TRIM56* overexpression plasmids (Fig. S4C, D). Notably, immunoblotting analysis showed that the overexpression of TRIM56 attenuated the reno-protective effect of *PVT1* ASO on mitochondrial function in the context of hyperglycemia (Fig. 4I). Concurrently, the results of OCR revealed that *TRIM56* siRNA ameliorated mitochondrial damage status of podocytes which was induced by HG treatment through increasing the mitochondrial respiration at basal levels, maximal levels and spare capacity, and this effect was accompanied by higher ATP production (Figs. 4J, S4E). Also, EPP revealed that the podocytes switched from quiescent to energetic metabolism due to *TRIM56* knockout (Fig. 4K). We also observed that the suppression of *TRIM56* mitigated the disruption of integrity of mitochondrial outer membrane and curtailed the cytosolic dsDNA signals when compared with HG group (Fig. 4L). The copy number of mtDNA released into the cytosol under hyperglycemia environment was reduced following *TRIM56* siRNA treatment (Fig. 4M). In addition, HG-induced activation of inflammatory factors was abolished due to silencing TRIM56 (Fig. 4N). Collectively, we concluded that the binding of *PVT1* to TRIM56 increased TRIM56 protein levels, and *PVT1*-induced TRIM56 promotion was sufficient to induce mitochondrial dysfunction and inflammation in podocytes.

### PVT1 enhances TRIM56 binding to AMPKα to promote its degradation via ubiquitin-dependent manner

**Fig. 5:**
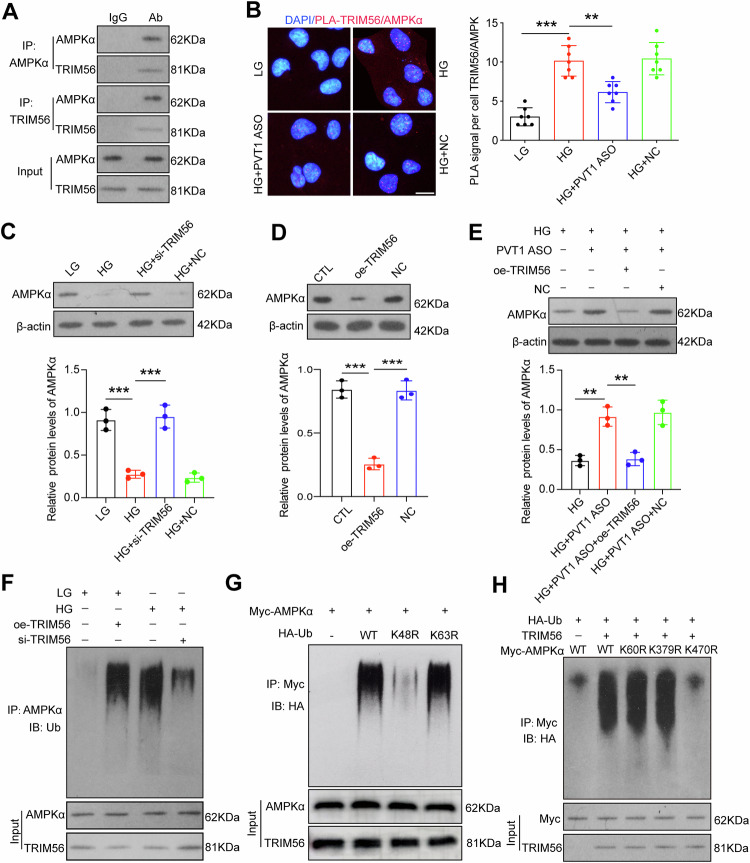
PVT1 enhances TRIM56 binding to AMPKα to promote its degradation via ubiquitin-dependent manner. **A** The interaction between endogenous TRIM56 and AMPKα in podocytes was evaluated by co-IP (*n* = 3). **B** Representative fluorescence images of PLA foci (red) represent the proximity of TRIM56 and AMPKα in podocytes (n = 7). Scale bar = 50 μm. **C** Expression of AMPKα in podocytes under different conditions (*n* = 3). **D** Western blotting detected the effect of TRIM56 on the expression of AMPKα in podocytes (n = 3). **E** Expression of AMPKα in podocytes under different conditions (*n* = 3). **F** The ubiquitination of AMPKα was evaluated in podocytes under different conditions (*n* = 3). **G** The ubiquitination of AMPKα was evaluated in the presence or absence of HA tagged WT or site-specific ubiquitin mutants of podocytes. **H** The ubiquitination of Myc-WT, -K60R, -K379R, or -K470R mutants of AMPKα was detected in podocytes under different conditions (*n* = 3). Error bars represent the mean± S.D, **P < 0.01, and ***P < 0.001.

Subsequently, we investigated the precise mechanism by which PVT1 modulates mitochondrial homeostasis disruption via TRIM56. By searching ProteinPrompt (http://proteinformatics.uni-leipzig.de/protein_prompt/), BioGRI (https://thebiogrid.org/123596), IntAct (https://www.ebi.ac.uk/legacy-intact/interactors/id:Q9BRZ2*) and STRING (https://string-db.org/network/9606.ENSP00000305161) databases, we found that AMPKα protein, which plays a major role in mitochondrial homeostasis, may be one of the possible candidates for binding to TRIM56. Then, we performed co-immunoprecipitation (coIP) assay to confirm the interaction of TRIM56 with AMPKα in podocytes (Fig. 5A). By using in situ proximity ligation assay (PLA), we found that the foci of TRIM56/AMPKα, which indicate the endogenous binding between TRIM56 and AMPKα, was overtly enhanced by HG treatment in podocytes, and *PVT1* ASO decreased their interaction (Fig. 5B). The expression of AMPKα was decreased after HG treatment, while transfection of si-*TRIM56* maintained AMPKα protein levels (Fig. 5C), and the overexpression of TRIM56 was accompanied by a significant reduction in the protein abundance of AMPKα in podocytes (Fig. 5D). Concomitantly, suppression of *PVT1* by ASO restored the protein levels of AMPKα under hyperglycemia conditions, in contrast overexpressed TRIM56 abolished the effect of *PVT1* ASO on AMPKα (Fig. 5E). These findings suggest that *PVT1* reduces AMPKα via upregulation of TRIM56.

Increasing amount of evidence suggest that members of the TRIM family, one of the largest families of RING-finger domain-containing E3 ligases, mediate posttranslational modifications of many proteins [18]. Indeed, the ubiquitination of AMPKα was markedly enhanced in the context of hyperglycemia when compared to LG group in podocytes, knockdown of TRIM56 in podocytes attenuated HG-induced AMPKα ubiquitination, while the overexpression of TRIM56 promoted the ubiquitination of AMPKα (Fig. 5F), suggesting that ubiquitin-dependent degradation of AMPKα may be associated with E3 ubiquitin ligase TRIM56. Given that multiple sites existing in ubiquitin give rise to substrate ubiquitination, we examined the specific site of ubiquitination involved. By constructing two mutants of ubiquitin (lysine 48 or lysine 63 to arginine [K48R or K63R, respectively]), we identified that TRIM56-induced AMPKα ubiquitination occurred through K48-linked chains rather than K63-linked chains (Fig. 5G). Next, we mutated three potential ubiquitination sites on AMPKα and found that among the three mutants (K470R, K60R and K379R), only AMPKα K470R ubiquitination was affected by TRIM56 and mutation of K470R weakened the role of TRIM56 in mediating AMPKα degradation (Fig. 5H). Thus, TRIM56 preferentially catalyzes polyubiquitination of AMPKα at K470 leading to its degradation.

### *TRIM56* deficiency reversed podocytes injury and restored the AMPKα expression of glomeruli in STZ-induced diabetic mice

**Fig. 6:**
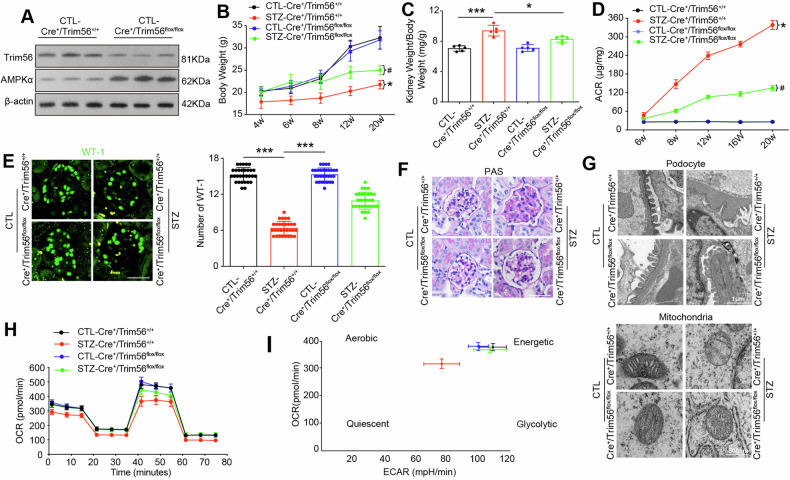
*TRIM56* deficiency reversed podocytes injury and restored the AMPKα expression of glomeruli in STZ-induced diabetic mice. **A**. Expression of Trim56 and AMPKα were detected by western blotting in glomerulus isolated from *Cre*^*+*^*/Trim56*^*flox/flox*^ mice and littermate controls (*n* = 3). **B** Temporal changes in body weights of the mice were measured respectively at 4 weeks (*n* = 6), 6 weeks (*n* = 6), 8 weeks (*n* = 6), 12 weeks (*n* = 6), and 20 weeks (*n* = 5) after DKD initiation. *P < 0.05 vs CTL-*Cre*^*+*^*/Trim56*^*+/+*^, ^#^*P* < 0.05 vs STZ-*Cre*^*+*^*/Trim56*^*+/+*^. **C** Kidney weight to body weight ratio was elevated in mice at 20 weeks after DKD initiation (*n* = 5). **D** Temporal ACR of mice were determined respectively at 6 weeks (*n* = 6), 8 weeks (*n* = 6), 12 weeks (*n* = 6), 16 weeks (*n* = 6), and 20 weeks (*n* = 5). *P < 0.05 vs CTL-*Cre*^*+*^*/Trim56*^*+/+*^, ^#^*P* < 0.05 vs STZ-*Cre*^*+*^*/Trim56*^*+/+*^. **E** Representative fluorescence images of WT-1 in glomeruli isolated from the four groups at 20 weeks after DKD initiation (*n* = 5). Scale bar = 50 μm. **F** PAS staining of glomeruli from the four groups at 20 weeks after DKD initiation (*n* = 5). Scale bar = 50 μm. **G** TEM image of podocytes, GBM, and mitochondria in primary podocytes from the four groups at 20 weeks after DKD initiation (*n* = 5). **H** Measurement of the mitochondrial OCR of primary podocytes from the four groups at 20 weeks after DKD initiation (*n* = 5). **I** Energy phenotype profile (EPP) was exhibited. Error bars represent the mean± S.D, *P < 0.05, **P < 0.01, and ***P < 0.001.

Next, we examined the podocyte-specific role of TRIM56 in diabetic mice by generating podocyte-specific *Trim56* knockout mice. We crossed *Nphs2-Cre* and *Trim56*^*flox/flox*^ mice to generate *Nphs2-Cre/Trim56*^*flox/flox*^ (*Cre*^*+*^*/Trim56*^*flox/flox*^) mice (Fig. S5A), which was confirmed by tail genotyping (Fig. S5B).| Immunofluorescence analysis of kidney sections(Fig. S5C) and western blot analysis of isolated glomeruli (Fig. 6A) confirmed the reduction of Trim56 protein levels in the knockout mice. We also found higher levels of AMPKα protein in the isolated glomeruli from *Cre*^*+*^*/Trim56*^*flox/flox*^ mice than control mice by western blotting analysis (Fig. 6A). Next, we used STZ to induce diabetes in these mice (Figure S5D), and found that there were no significant differences in blood glucose levels between the STZ-*Cre*^*+*^*/Trim56*^*+/+*^ and STZ-*Cre*^*+*^*/Trim56*^*flox/flox*^ groups at all time point (Fig. S5E). As shown in Fig. 6B, STZ-*Cre*^*+*^*/Trim56*^*+/+*^mice had lower body weight as compared with the non-diabetic counterparts after 6 weeks of diabetes, while the STZ-*Cre*^*+*^*/Trim56*^*flox/flox*^ mice showed improved growth compared to STZ-*Cre*^*+*^*/Trim56*^*+/+*^ mice. The kidney weight to body weight ratio in the STZ-*Cre*^*+*^*/Trim56*^*flox/flox*^ mice was decreased compared with that in the STZ-*Cre*^*+*^*/Trim56*^*+/+*^ group at 20 weeks after induction of diabetes (Fig. 6C). Importantly, STZ-*Cre*^*+*^*/Trim56*^*flox/flox*^ mice exhibited a notable decrease in proteinuria when compared with STZ-*Cre*^*+*^*/Trim56*^*+/+*^ mice after 8 weeks of diabetes (Fig. 6D). And compared with CTL-*Cre*^*+*^*/Trim56*^*+/+*^ mice, the STZ-*Cre*^*+*^*/Trim56*^*flox/flox*^ mice exhibited decreased staining for WT-1, while knockout of *Trim56* restored the expression(Fig. 6E). Morphological experiments (PAS, and TEM) showed the diabetic glomerulopathy, which was evidenced by GBM thickening, and podocyte foot process broadening and effacement in STZ-*Cre*^*+*^*/Trim56*^*+/+*^ mice, all of which were ameliorated in *Trim56* knockout mice (Figs. 6F–G, S5F).

In addition, compare with STZ-*Cre*^*+*^*/Trim56*^*+/+*^ mice, the mitochondrial damage evidenced by mitochondrial swelling, fragmentation and vacuolization was attenuated in primarily cultured podocytes from STZ-*Cre*^*+*^*/Trim56*^*flox/flox*^ mice (Fig. 6G). Consistently, OCR analysis in these primarily cultured podocytes revealed that, the mitochondrial respiratory functions were ameliorated in STZ-*Cre*^*+*^*/Trim56*^*flox/flox*^ mice at basal and maximal levels, and the production of ATP was also increased, when compared with STZ-*Cre*^*+*^*/Trim56*^*+/+*^ mice (Figs. 6H, S5G). EPP revealed that the primary podocytes from STZ-*Cre*^*+*^*/Trim56*^*+/+*^ mice were switched from energetic to quiescent metabolism, while the podocytes from Trim56 knockout mice were changed back to energetic metabolism (Fig. 6I). Collectively, our results suggested that deletion of TRIM56 in podocytes mitigate podocyte and glomerular injury.

### TRIM56-mediated podocytes mitochondrial dysfunction and inflammation via AMPKα in diabetic kidney disease

**Fig. 7:**
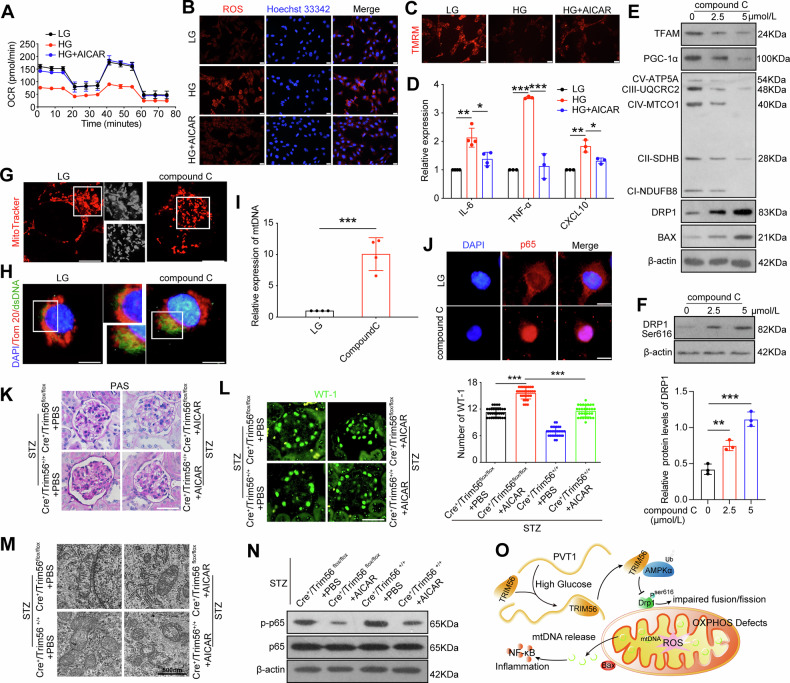
TRIM56-mediated podocytes mitochondrial dysfunction and inflammation via AMPKα in diabetic kidney disease. **A**. Measurement of the mitochondrial OCR of podocytes under different conditions (*n* = 3). **B** Representative fluorescence images of mtROS in podocytes (*n* = 3). Scale bar = 50 μm. **C** Representative fluorescence images of ΔΨm in podocytes (*n* = 3). Scale bar =50 μm. **D** Relative mRNA levels of *IL-6* (*n* = 4)*, TNF-α* (*n* = 3), and *CXCL10* (*n* = 3) in podocytes cultured under the indicated conditions. **E** Expression of TFAM, PGC-1α, BAX, DRP1, and mitochondrial OXPHOS proteins in podocytes with different concentration of compound C (*n* = 3). **F** Expression of DRP Ser616 in podocytes with different concentration of compound C (*n* = 3). **G** Representative fluorescence images of MitoTracker in podocytes (*n* = 3). Scale bar =50 μm. **H** Representative fluorescence images of podocytes cultured under the indicated conditions with anti-Tom 20 (red) and anti-dsDNA (green) antibodies (*n* = 3). Scale bar =50 μm. **I** Levels of *mtDNA* in cytoplasm of podocytes under different conditions (*n* = 4). **J** Representative fluorescence images of podocytes with anti-p65 antibody (red) (*n* = 3). Scale bar =50 μm. **K** PAS staining of glomeruli from the four groups after AICAR injection for 14 weeks (*n* = 5). Scale bar = 50 μm. **L** Representative fluorescence images of WT-1 in glomeruli isolated from the mice after AICAR injection for 14 weeks (*n* = 5). Scale bar = 50 μm. **M** TEM revealed mitochondrial morphological changes in primary podocytes from the four groups after AICAR injection for 14 weeks (*n* = 5). **N** Western blotting demonstrated the expression of p-p65, p65 in primary podocytes from the four groups after AICAR injection for 14 weeks (*n* = 3). **O** Schematic model of the role of *PVT1*. Under hyperglycemia conditions, PVT1 was upregulated. Increased PVT1 paticipated in mitochondrial dysfunction by interacting with TRIM56 post-transcriptionally to regulate the ubiquitination of AMPKα, which resulted abnormal mitochondrial biogenesis and fission. In addition, the cytosolic mtDNA, released from damaged mitochondria, linking mitochondrial homeostasis disruption to inflammatory responses in podocytes. Error bars represent the mean± S.D, *P < 0.05, **P < 0.01, and ***P < 0.001.

Firstly, we cultured podocytes by high glucose and applied carboxamide ribonucleotide (AICAR), an AMPKα pharmacological activator, to explore the role of AMPKα in mitochondrial dysfunction and inflammation of podocytes under high glucose conditions (Fig. S6A). OCR analysis revealed that AMPKα activator AICAR protected mitochondrial function at basal respiration, maximal respiration, and spare respiratory capacity (Figs. 7A, S6B), and the EPP of podocytes changed from quiescent metabolism to energetic metabolism due to AICAR treatment (Fig. S6C). Meanwhile, we found mtROS accumulation induced by HG stimulation was suppressed in the presence of AICAR in podocytes (Fig. 7B). As exhibited in Fig. 7C, a pronounced abatement of fluorescence intensity of TMRM in hyperglycemia-treated podocytes reflected the loss of mitochondrial membrane potential (ΔΨm), while ΔΨm was increased in the presence of AICAR. In addition, the increased expression of inflammation-related molecules under HG conditions were substantially reversed by AICAR (Fig. 7D).

The balance between mitochondrial biogenesis, fission and fusion, and mitophagy-the selective elimination of non-functional and damaged mitochondria manipulates the homeostasis of mitochondria [19]. Hence, we examined the influence of AMPKα on mitochondrial biogenesis and dynamics in the presence of AMPKα inhibitor compound C (Fig. S6D). In contrast to what was observed in HG group, suppression of AMPKα lead to an evident reduction of OXPHOS-related proteins and PGC-1α, a prominent regulator to control mitochondrial biogenesis, as well as an augmentation of DRP1 in a dose-dependent manner (Fig. 7E). Moreover, DRP1 function is mediated by several posttranslational modifications including phosphorylation, Ser616 phosphorylation (pro-fission site) is linked to increased activity of DRP1, whereas Ser637 phosphorylation (anti-fission site) is related to reduced activity [20]. We found that the using of compound C increased Ser616 phosphorylation levels in a dose-dependent manner (Fig. 7F). Besides, excessive fragmentation of mitochondria in podocytes was observed in compound C group (Fig. 7G), and the treatment of compound C disrupted the integrity of mitochondrial outer membrane and significantly increased the cytosolic dsDNA signals in podocytes (Fig. 7H, I). Furthermore, immunofluorescence revealed that compound C led to increased nuclear translocation of the p65 subunit of NF-κB (Fig. 7J). Taken together, our results indicated that AMPKα inhibits TRIM56-induced mitochondrial dysfunction and inflammation both in vivo and in vitro.

Following this, we investigated the involvement of AMPKα in Trim56-induced mitochondrial dysfunction and inflammation stimulated by STZ injection in vivo. The STZ-*Cre*^*+*^*/Trim56*^*+/+*^ mice and STZ-*Cre*^*+*^*/Trim56*^*flox/flox*^ mice were received daily intraperitoneally injections with 0.5 mg/g AICAR or vehicle (PBS) at 16 weeks of age (6 weeks after DKD initiation), and this injection would last for 14 weeks (Fig. S6E), the expression of AMPKα was determined by western blotting (Fig. S6F). PAS staining showed that the degree of glomerular injury was significantly improved after injection of AICAR (Fig. 7K), the relative mesangial areas (%) and glomerular tuft volume were calculated (Fig. S6G). And compared to the PBS control groups, the increase in the expression of podocyte specific marker proteins WT-1 were determined by immunofluorescence (Fig. 7L). In addition, TEM showed that mitochondrial swelling, fragmentation and vacuolization were attenuated in primary podocytes after injection of AICAR (Fig. 7M). Also, we found that the treatment of mice with AICAR induced marked inhibition of NF-κB pathway in primary podocytes (Fig. 7N). These results confirmed the involvement of AMPKα in TRIM56-induced cellular metabolic defects and inflammatory response in diabetic kidney disease.

## Discussion

Here, in the current study, we evidenced that *PVT1* plays a pivotal role in mitochondrial defect and inflammation of podocytes elicited by hyperglycemic condition through exerting the promotive effects on TRIM56 post-translational expression in vivo and in vitro. The upregulation of TRIM56 was shown to remarkably induce ubiquitination of AMPKα, thereby inhibiting PGC-1α and phosphorylating DRP1 Ser616 to induce abnormal mitochondrial biogenesis and fission, and subsequently the aberrant translocation of mtDNA induced by mitochondrial dysfunction activated NF-κB likely via activation of cGAS-STING pathway as shown previously [14, 21], linking mitochondrial metabolic insufficiency to enhanced inflammatory cytokines expression in podocytes (Fig. 7O). Our findings provide plausible evidence for *PVT1* in DKD pathogenesis and progression.

Recently, voluminous numbers of lncRNA transcripts were unveiled by deploying microarray analysis and cDNA library sequencing. Much has been learned about that lncRNAs can exert their functions by modulating the epigenetic state of protein-coding genes via cis- and trans-acting mechanisms, involving in mRNA translation and stability, as well as conducting organelle biogenesis and subcellular trafficking, thereby to regulate cell biology, such as cell proliferation, differentiation, migration and/or invasion [22–25]. So far, it is well perceived that the susceptibility to develop ESRD in diabetes is affected by genetic components since several lncRNAs have been clearly explicated in the initiation and progression of DKD [26–29]. Among them, *PVT1* gene was reported to be potently correlated with DKD in the Pima Indians, a group with the highest prevalence of T2DM in the world, by Genome-wide analysis [12]. Additionally, Alvarez et al. found that *PVT1* may intervene DKD development and progression [30]. Consistent with the investigations above, we found *PVT1* was stimulated in podocytes treated with high glucose and from diabetic mice.Also, high expression of *PVT1* was positively correlated with the level of proteinuria and Scr in patients with DKD. We acknowledged the correlation may be not strong, which due to our small sample size. In our future studies, the sample size of each group will be increased for further verifying our findings.

Given the subcellular localization of PVT1 in podocytes, we spectuated that PVT1 may function by interacting with RNA-binding proteins (RBPs). By performing RNA pull-down assay and searched MitoCarta3.0 datasets, we selected TRIM56, a member of TRIM superfamily proteins, as a protein partner of PVT1. Many members of the TRIM superfamily proteins contribute to various biological processes that are associated with innate immunity [31]. The highly conserved N-terminal RBCC (N-terminal RING finger/B-box/coiled coil) motif, which defines characteristic of this superfamily, comprises a RING domain, one or two B-box domains and a coiled-coil domain. Based on differences in C-terminal domain composition, the classification of human TRIM protein subfamilies denoted C-I to C-IX [32]. Notably, some of them partake in antiviral innate immune responses in a ubiquitination-dependent manner. For example, Trim25, belongs to the C-IV family, induces the Lys 63-linked ubiquitination of RIG-I to regulate innate immunity to viral infection [33]. However, the role of TRIM protein in the progression of DKD was unveiled. Our study delineated that TRIM56, belongs to the C-V family, functions as an E3 ligase to catalyze the hyperglycemia-induced ubiquitination of AMPKα, thereby regulating mitochondrial biology and homeostasis to influence mtDNA leakage. Another surprising finding in our study is that the spare respiratory capacity of podocytes was significantly increased on account of *TRIM56* depletion, indicating that inhibiting *TRIM56* not only improves mitochondrial function of podocytes, but also enhances their metabolic plasticity and adaptability.

In this study, by searching ProteinPrompt, BioGRI, IntAct and STRING databases, we found that AMPKα protein, may be one of the possible candidates for binding to TRIM56. By performing a series of verification experiments, we confirmed that TRIM56 interacted with AMPKα and degraded its expression in a ubiquitin-dependent manner. More importantly, AMPK signaling has been implicated as a target for correcting metabolism and mitochondrial function, especially in the kidney. Mitochondrial biogenesis, increases OXPHOS and ATP production, is dominated regulated by PGC-1α, while inactivation of PGC-1α in the kidney reduces mitochondrial function and metabolism [34, 35]. In our study, PGC-1α deficiency resulted from the using of AMPKα inhibitor compound C notably decreased expression of proteins for OXPHOS and impaired mitochondrial function. Furthermore, it has been illustrated that endurance exercise-induced mitochondrial biogenesis promotes TFAM expression, subsequently increasing mitochondrial transcripts and function via protecting mtDNA from excess ROS and degradation [34, 36]. We also found that the reduction of PGC-1α was accompanied by the downregulation of TFAM in podocytes pretreated with compound C. Meanwhile, shapes and patterns of mitochondria, like fragmented, elongated and the number of organelles per area, transform promptly with different conditions and can differ vastly between cell kinds and disease states [37]. For instance, elongated shape of mitochondria could ensue from increased fusion, reduced fission, boosted growth or any amalgamation of these phenomena [38], while fission events, mainly mediated by conserved DRP1, facilitate engendering small fragments of mitochondria that can subsequently be earmarked for degradation by mitophagy [39]. In line with previous reports, our data shows the disruption of the balance between fusion and fission alters mitochondrial morphology and impairs mitochondrial function, the excessive mitochondrial fragmentation has been implicated as a key event in mitochondrial damage and kidney injury during DKD. In this context, AMPKα and phosphorylation of DRP1 were involved into mediating mitochondrial fission, however, the role of AMPKα in mitochondrial fission is still controversial. Toyama et al. showed that direct pharmacological activation of AMPK promoted mitochondrial fragmentation through phosphorylation of mitochondrial fission factor (MFF), a mitochondrial outer-membrane receptor for DRP1 [40]. Conversely, we demonstrated that the suppression of AMPKα by compound C increased total DRP1 expression and its phosphorylation at site Ser616, thereby promoting DRP1-mediated mitochondrial fission. In agreement with our finding, Wang Q illustrated that activation of AMPKα by metformin impinged mitochondrial fission and decreased endothelial cell apoptosis via the reduction of DRP1 expression in high glucose-stimulated endothelial cells [41]. In summary, as revealed, AMPKα serves as a principle mediator of the cellular response to energetic stress and orchestrates multiple facets of mitochondrial biology and homeostasis, including control of mitochondrial biogenesis, regulation of the shape of the mitochondrial network, and mitochondrial quality control through regulation of autophagy and mitophagy [42].

An accumulation of evidence proposes that mitochondria could serve as a principal hub of pro-inflammatory signaling to regulate inflammation during kidney injury [14, 36, 43, 44]. On the one hand, the fusion of mitochondria into linear or tubular networks confines detrimental mutations in mtDNA, triggers supercomplexes of the electron transport chain (ETC) maximizing OXPHOS activity [45], conversely, mitochondrial fragmentation decreases OXPHOS activity and induces mitochondrial outer membrane permeabilization (MOMP), facilitating BAX insertion and oligomerization in mitochondria, and in the circumstances, damage-associated molecular patterns including formyl peptides and mtDNA could be released into the cytosol through BAX pores in the mitochondrial outer membrane (OMM) [46], binding to Toll-like receptors or nucleotide-binding oligomerization domain-containing protein (NOD)-like receptors [47], and thereby triggering kidney inflammation. On the other hand, mtROS acts as stimulator of inflammation that increases pro-inflammatory gene expression, activating NF-κB signaling [48–50]. Consistent with this finding, our study demonstrated that damaged mitochondria in podocytes released mtDNA into the cytosol probably through BAX-activated pores in the OMM, resulting in inflammation manifected as increased expression of IL-6, ICAM-1, CCL2 and TNF-α. Besides, we used ethidium bromide (EtBr) to block the replication of mtDNA, and found that EtBr abolished the phosphorylation NF-κB p65 and decreased the release of proinflammatory cytokines. In addition, we found that the production of mtROS was significantly increased in the HG-treated podocytes, while the suppression of *PVT1* decreased mtROS levels. Hence, the induction of inflammation triggered by mitochondria damage in HG-treated podocytes may be an outcome of both mtDNA leakage and mtROS generation. Taken together, we spectuated that the cytosolic mtDNA, released from damaged mitochondria, linking mitochondrial homeostasis disruption to inflammatory responses in podocytes likely via activation of NF-κB pathway. However, TRIM56 can promote inflammatory responses through multiple molecular mechanisms. The mitochondrial dysfunction resulting release of mtDNA and mtROS triggered inflammation may only be one of the possibilities, which we demonstrated in the present study. Actually, TRIM56 itself can also modulate NF-κB and IFN pathways to trigger IFN response and inflammation [51, 52]. It is also reported that TRIM56 interacted with TAK1 and enhanced the M1-linked polyubiquitin chains of TAK1 which further potentiated the interaction between TAK1 and IKKα, thereby increasing the transcription of NF-κB downstream genes [53]. The results of our present study will provide evidence for elucidating the roles of TRIM56 in the progression of DKD.

In conclusion, despite the striking improvements in treatment efficacy and continuous broadening of knowledge on the pathogenesis of DKD, the concrete mechanism involved in DKD still remains challenging. Our study revealed the vital role of PVT1 in mitochondrial dysfunction and inflammatory responses of DKD, which may provide novel, more specific and less toxic therapeutic strategy for the treatment of DKD.

## Supplementary information

Supplementary Materials

## Data Availability

No publicly available data or shared data are cited. All data needed to evaluate the conclusion of the current study are present in the paper and/or the supplementary materials. Additional data are available from the corresponding author on request.
