# Supplementary material for: LncRNA PVT1 induces mitochondrial dysfunction of podocytes via TRIM56 in diabetic kidney disease
Source: Cell Death Dis. 2024 Sep 30;15(9):697. doi: 10.1038/s41419-024-07107-5 (PMC11442824; doi:10.1038/s41419-024-07107-5)

Supplementary Figures

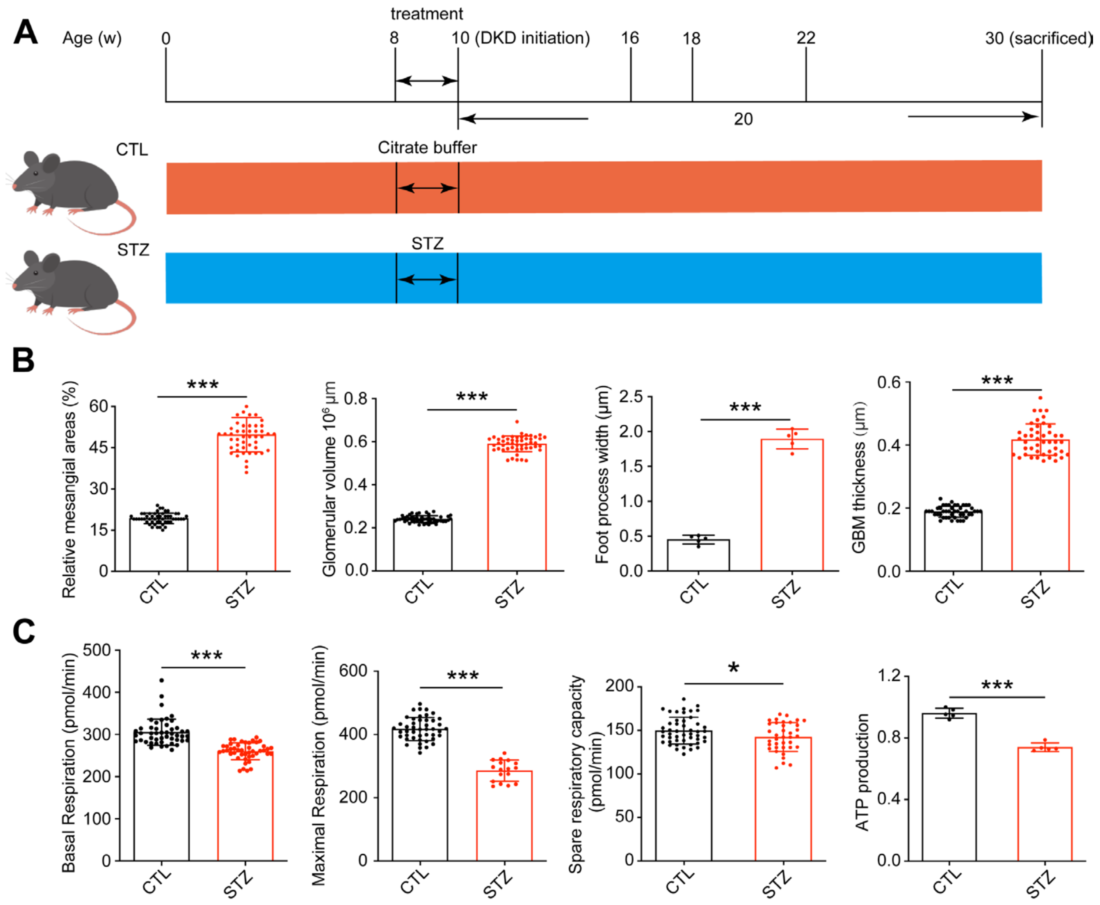

Figure S1 **A** Schematic diagram for the DKD modeling protocol. **B** The relative mesangial area (%), glomerular volume, foot process width, GBM thickness were analyzed (n = 5). **C** Basal respiration level, Maximal respiration level, Spare respiratory capacity and ATP production was analyzed. Error bars represent mean ± S.D, $^{*}P < 0.05$, $^{**}P < 0.01$, and $^{***}P < 0.001$.

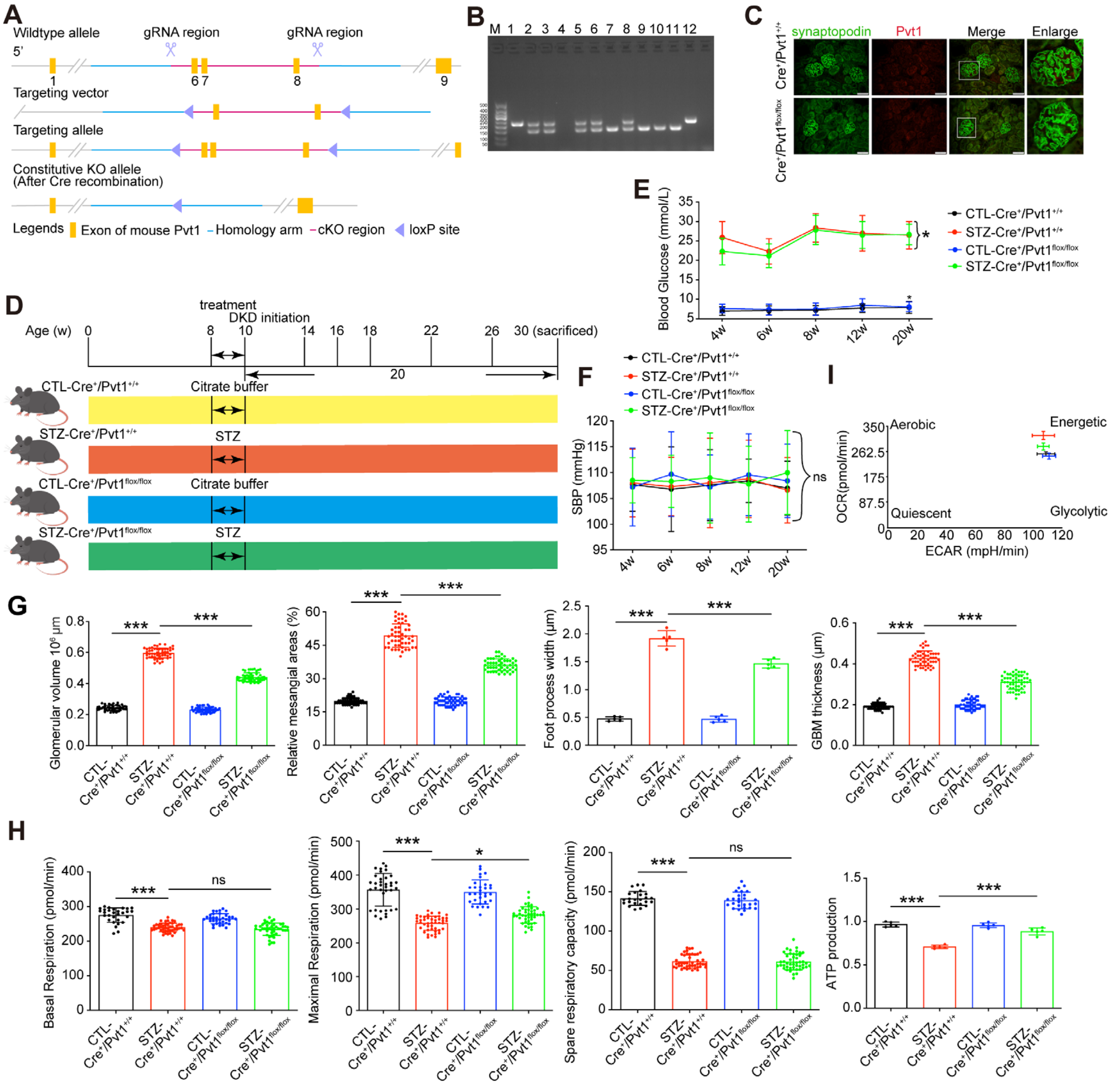

Figure S2

A Gene targeting strategy. **B** Genotyping of offsprings by PCR showing WT (146 bp), heterozygous (215 bp and 146 bp), and homozygous (215 bp) alleles. **C** *Pvt1* expression was detected by RNA-FISH in glomerulus isolated from $Cre^{+}/Pvt1^{flox/flox}$ mice and littermate controls (n = 3). **D** Schematic diagram for the DKD modeling protocol. **E** Temporal blood glucose of mice were detected respectively at 4 weeks (n = 6), 6 weeks (n = 6), 8 weeks (n = 5), 12 weeks (n = 5), and 20 weeks (n = 5) after DKD initiation. $^{*}P < 0.05$ vs CTL-$Cre^{+}/Pvt1^{+/+}$. **F** Temporal SBP of mice were detected respectively at 4 weeks (n = 6), 6 weeks (n = 6), 8 weeks (n = 5), 12 weeks (n = 5), and 20 weeks (n = 5) after DKD initiation. **G** The glomerular volume, relative mesangial area, foot process width, GBM thickness was analyzed (n=5). **H** Basal respiration level, Maximal respiration level, Spare respiratory capacity, and ATP production was analyzed (n = 5).

I Energy phenotype profile (EPP) was exhibited. Error bars represent the mean ± S.D, $^{*}P < 0.05$, and $^{***}P < 0.001$.

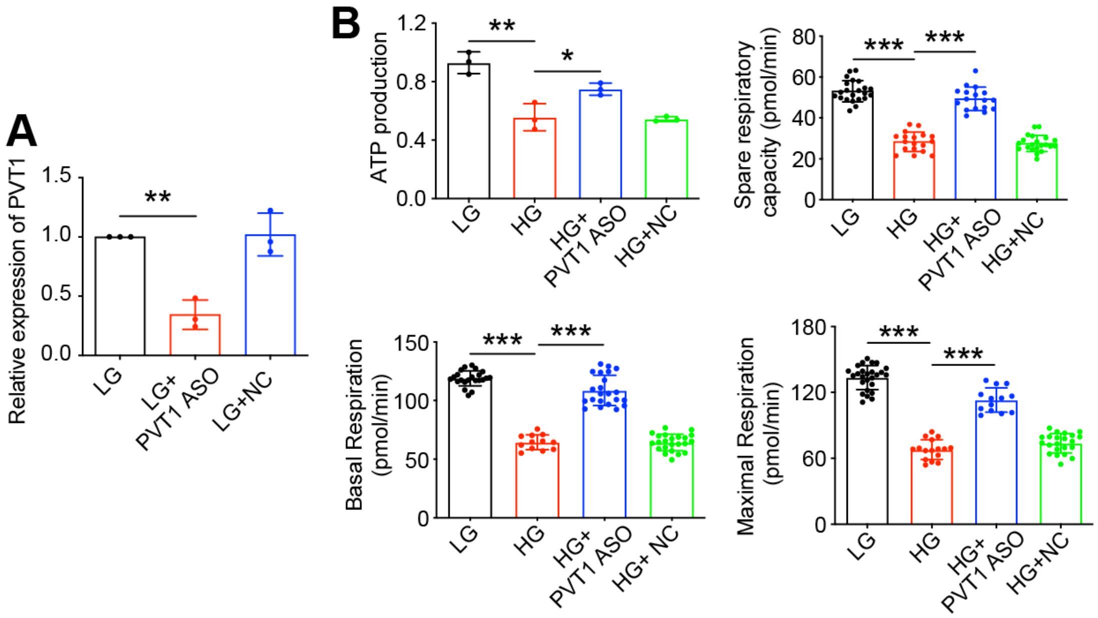

Figure S3 A The efficiency of *PVT1*-ASO was confirmed by qRT-PCR (n = 3). **B** ATP production, Spare respiratory capacity, Basal respiration level, and Maximal respiration level were analyzed (n = 3). Error bars represent the mean± S.D, $^{*}P < 0.05$, $^{**}P < 0.01$, and $^{***}P < 0.001$.

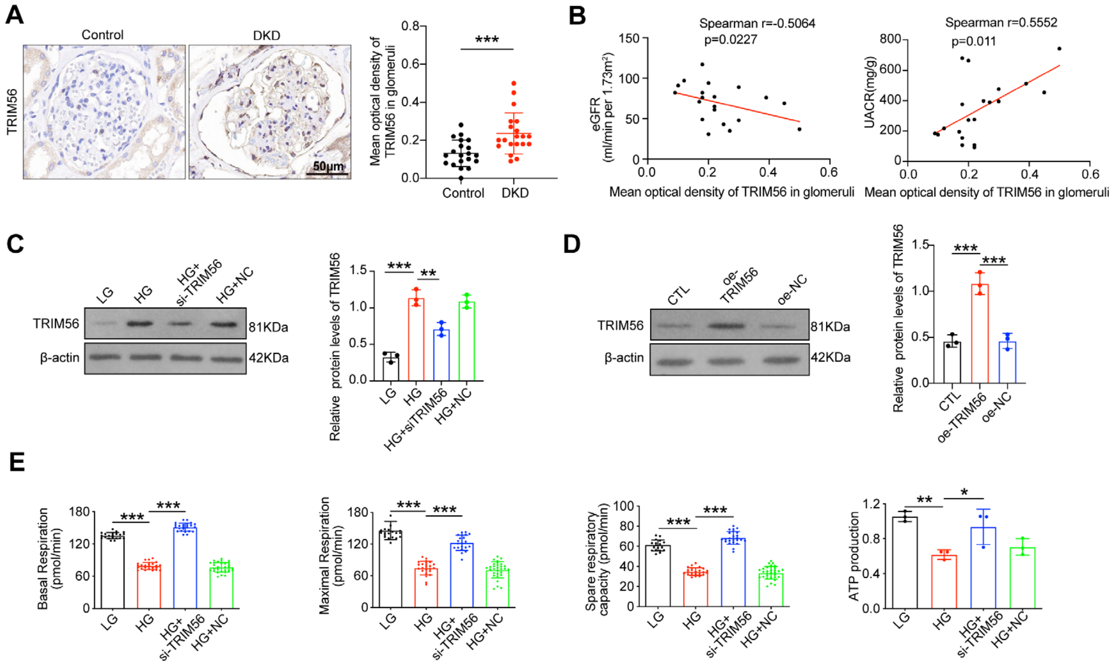

Figure S4 A. Representative images of TRIM56 immunohistochemical staining in human kidney tissues from DKD and control patients. Scale bar = 50 μm. **B**. The correlation of glomerular TRIM56 expression with estimated glomerular filtration rate (eGFR), and UACR. n=20. **C** Expression of TRIM56 in podocytes was detected using western blotting (n = 3). **D** The efficiency of oe-*TRIM56* was confirmed by western blotting (n = 3). **E**. Basal respiration level, Maximal respiration level, Spare respiratory capacity and ATP production was analyzed. Error bars represent mean ± S.D, $^{*}P < 0.05$, $^{**}P < 0.01$, and $^{***}P < 0.001$.

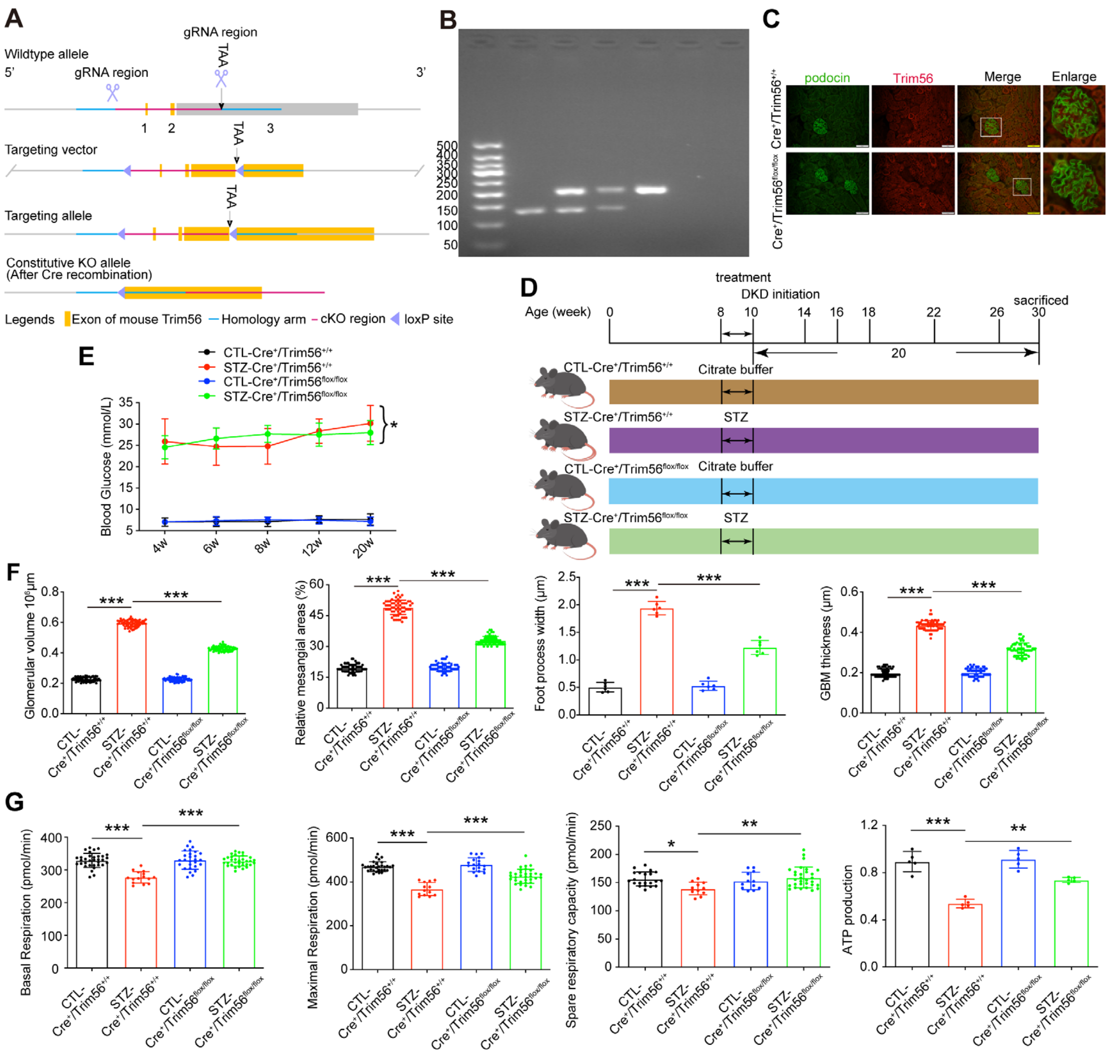

Figure S5 A Gene targeting strategy. **B** Genotyping of off springs by PCR showing WT (129 bp), heterozygous (198 bp and 129 bp), and homozygous (198 bp) alleles. **C** *Trim56* expression was detected by immunofluorescence in glomerulus isolated from *Cre*$^{+}$*/Trim56*$^{flox/flox}$ mice and littermate controls (n = 3). **D**. Schematic diagram for the DKD modeling protocol. **E** Temporal blood glucose of mice were detected respectively at 4 weeks (n = 6), 6 weeks (n = 6), 8 weeks (n = 6), 12 weeks (n = 6), and 20 weeks (n

= 5) after DKD initiation. $^{*}P < 0.05$ vs CTL-*Cre*$^{+}$/*Trim56*$^{+/+}$. **F** The glomerular volume, relative mesangial area, foot process width, and GBM thickness was analyzed (n = 5). **G**. Basal respiration level, Maximal respiration level, Spare respiratory capacity, and ATP production was analyzed. Error bars represent mean ± S.D, $^{*}P < 0.05$, $^{**}P < 0.01$, and $^{***}P < 0.001$.

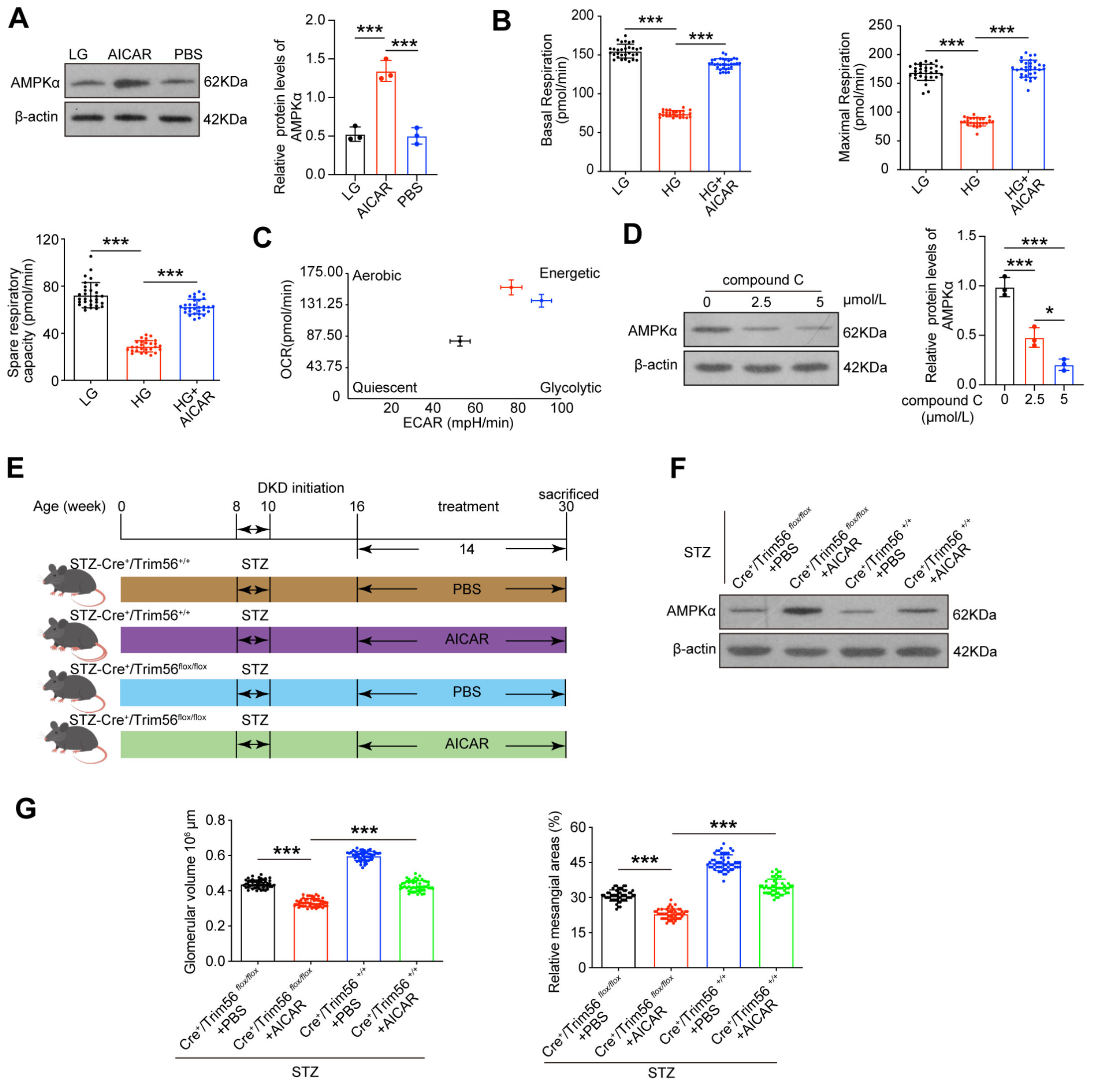

Figure S6

A Expression of AMPKα in podocytes in the presence of AICAR was detected by western blotting (n = 3). Schematic diagram for the DKD modeling protocol. **B** Basal respiration level, Maximal respiration level, and Spare respiratory capacity were analyzed (n = 3). **C** Energy phenotype profile (EPP) was mentioned. **D** Expression of AMPKα in podocytes in the presence of compound C was detected by western blotting (n = 3). **E** Schematic diagram for the DKD modeling protocol. **F** Expression of AMPKα

was detected by western blotting in glomerulus (n = 3). **G** The glomerular volume and relative mesangial area was qualified (n = 5). Error bars represent the mean± S.D, $^{***}P < 0.001$.

Supplemental Tables

Table S1. Primers for real-time PCR.

Gene	Primer	Sequence
PVT1 (Homo)	Forward	5'-CCGACTCTTCCTGGTGAAGC-3'
	Reverse	5'-GTATGGTCAGCTCAAGCCCA-3'
Pvt1 (Mus)	Forward	5'-TGGTAGGAGACAGACTTGCTCAGG-3'
	Reverse	5'- TAGTGCGGAGGAAGGCTGCTC-3'
BAX (Homo)	Forward	5'-GAGGTCTTTTTCCGAGTGGCA-3'
	Reverse	5'-GGCAAAGTAGAAAAGGGCGAC-3'
Tfam (Mus)	Forward	5'-GGGAATGTGGAGCGTGCTAA-3'
	Reverse	5'-GATAGACGAGGGGATGCGAC-3'
mt-Co1 (Mus)	Forward	5'-TCTGTTCTGATTCTTTGGGCACC-3'
	Reverse	5'-CTACTGTGAATATGTGGTGGGCT-3'
mt-Rnr2 (Mus)	Forward	5'-TCACCAGTCAAAGCGAACTAC-3'
	Reverse	5'-AAACGAACCTTTAATAGCGGC-3'
mt-Nd6 (Mus)	Forward	5'-CGCAAACAAAGATCACCCAGC-3'
	Reverse	5'-ATGTTGGAAGGAGGGATTGGG-3'
mt-Cytb (Mus)	Forward	5'-TCTACGCTCAATCCCCAATAAAC-3'
	Reverse	5'-GGCTTCGTTGCTTTGAGGTAT-3'
Cox4 (Mus)	Forward	5'-GAGAGCTTCGCCGAGATGAA-3'
	Reverse	5'-AATCAGAACGAGCGCAGTGA-3'
Uqcrc2 (Mus)	Forward	5'-CTCAAAGTTGCCCCGAAGGT-3'
	Reverse	5'-CCGATTCTTGACAGAGGAGCA-3'
Ndufb8 (Mus)	Forward	5'-ATGTTGCCGGGGTCATATCC-3'
	Reverse	5'-ATCGGGGTATGGCTCGTAGT-3'
Sdhb (Mus)	Forward	5'-GCAGTTTCAGGCCTGTCGAG-3'
	Reverse	5'-GGTCCCATCGGTAAATGGCA-3'
Il6 (Mus)	Forward	5'-CTTCTTGGGACTGATGCTGGT-3'
	Reverse	5'-CTCTGTGAAGTCTCCTCTCCG-3'
IL6 (Homo)	Forward	5'-CAATGAGGAGACTTGCCTGGT-3'
	Reverse	5'-GCAGGAACTGGATCAGGACT-3'
Tnf-α (Mus)	Forward	5'-AGCCGATGGGTTGTACCTTG-3'
	Reverse	5'-ATAGCAAATCGGCTGACGGT-3'

TNF-α (Homo)	Forward	5'-GAGGCCAAGCCCTGGTATG-3'
	Reverse	5'-CGGGCCGATTGATCTCAGC-3'
Cxcl10 (Mus)	Forward	5'-TGAGAATGAGGGCCATAGGGA-3'
	Reverse	5'-CAATGATCTCAACACGTGGGC-3'
CXCL10 (Homo)	Forward	5'-TGCCATTCTGATTTGCTGCC-3'
	Reverse	5'-TGCAGGTACAGCGTACAGTT-3'
Icam1 (Mus)	Forward	5'-CAAAGCTCGACACCCCTGAC-3'
	Reverse	5'-GTTTGTGCTCTCCTGGGTCG-3'
tRNA-LeuUUR (Homo)	Forward	5'-CACCCAAGAACAGGGTTTGT-3'
	Reverse	5'-TGGCCATGGGTATGTTGTTA-3'
β2-microglobulin (Homo)	Forward	5'-TGCTGTCTCCATGTTTGATGTATCT-3'
	Reverse	5'-TCTCTGCTCCCCACCTCCAAGT-3'
β-actin (Mus)	Forward	5'-GGCTGTATTCCCCTCCATCG-3'
	Reverse	5'-CCAGTTGGTAACAATGCCATGT-3'
β-actin (Homo)	Forward	5'-GAAGAGCTACGAGCTGCCTGA-3'
	Reverse	5'-CAGACAGCACTGTGTTGGCG-3'

Table S2. Characteristics of the clinical subjects.

Characteristics	Healthy controls (n = 25)	DKD patients (n = 47)	P value
Age, years	53.08 ± 11.03	57.74 ± 9.19	0.576
Men, %	48.00	55.32	0.624
HbA1c, %	5.57 ± 0.71	8.46 ± 1.33	< 0.001***
ACR, mg/g	1.78 (1.14-8.91)	373.5 (137.15-500.67)	< 0.001***
Serum creatine (Scr), μmol/L	62.00 (54.50-74.00)	114.00 (85.00-151.00)	<0.001***
BUN, mmol/L	4.87 ± 0.94	7.94 ± 3.51	<0.001***
eGFR, ml/min/1.73 m^2	106.72 ± 6.12	66.68 ± 21.20	< 0.001***
Total cholesterol, mmol/L	4.58 ± 0.87	4.78 ± 0.76	0.575
Triglycerides, mmol/L	1.49 (1.30-1.59)	2.15 (1.89-2.39)	< 0.001***
HDL-c, mmol/L	1.36 (1.28-1.54)	1.13 (0.96-1.34)	0.003**
LDL-c, mmol/L	2.64 (2.38-2.86)	2.99 (2.35-3.20)	0.076

*$P < 0.05$, **$P < 0.01$, ***$P < 0.001$.

Figure 2

2G

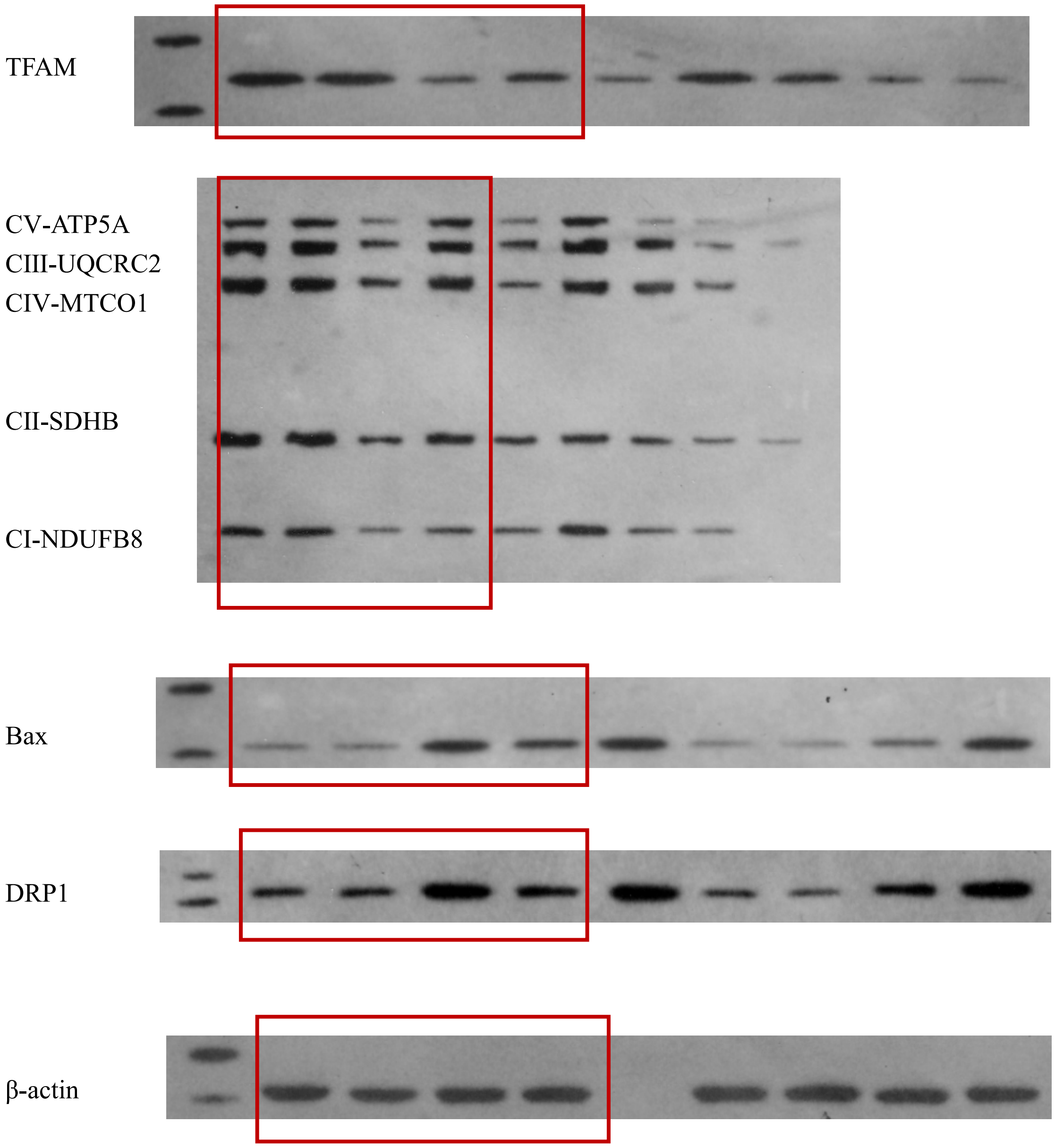
TFAM
CV-ATP5A
CIII-UQCRC2
CIV-MTCO1
CII-SDHB
CI-NDUFB8
Bax
DRP1
β-actin

Figure 3

3H

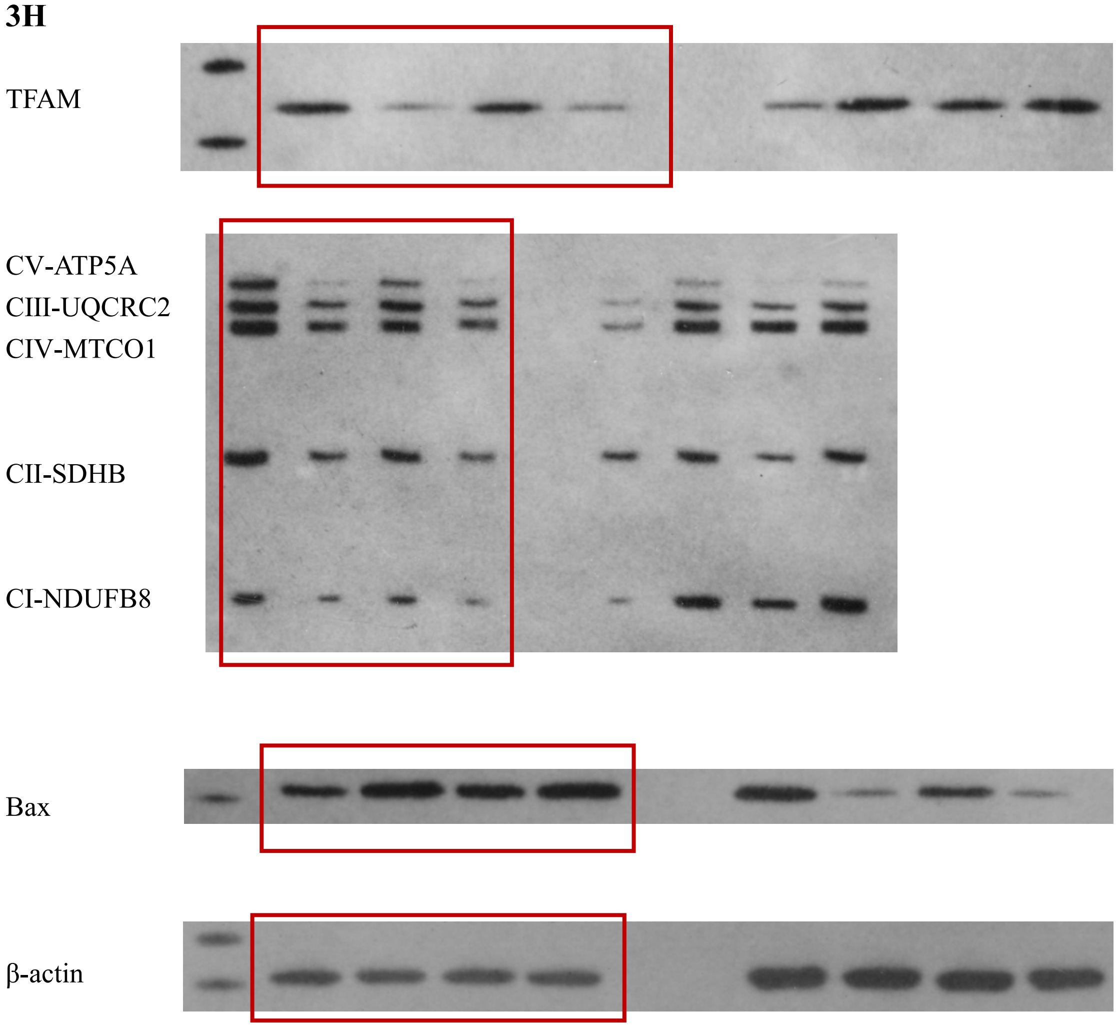

3I

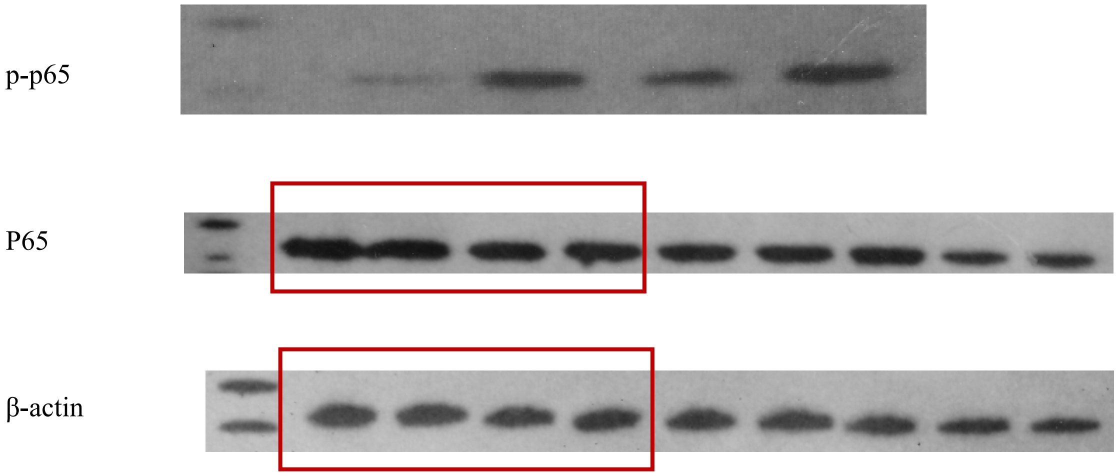

3K

p-p65

p65

β-actin

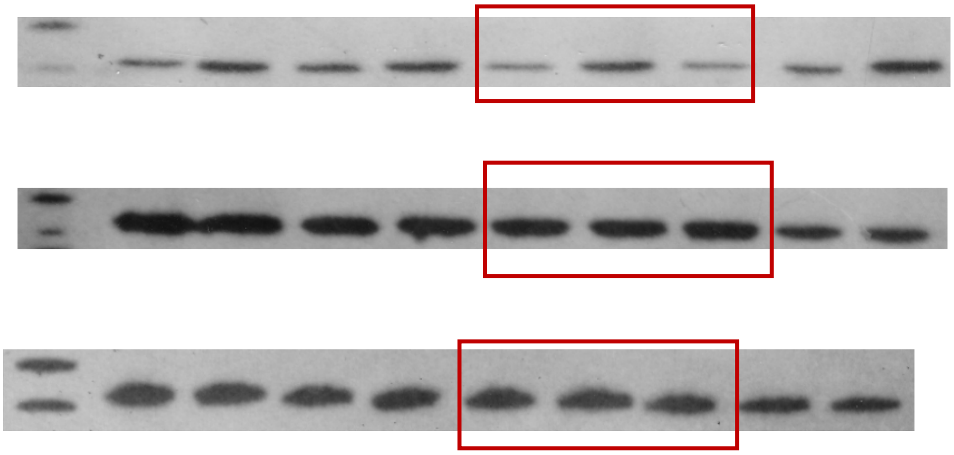

Figure4

4B

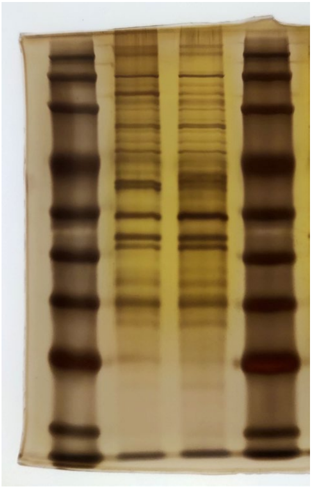

4C

TRIM56

β-actin

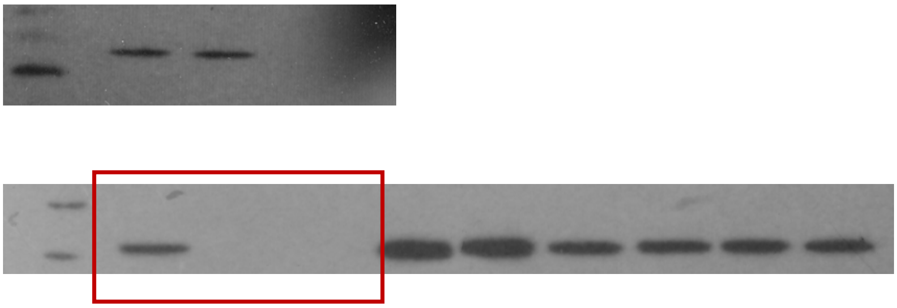

4E

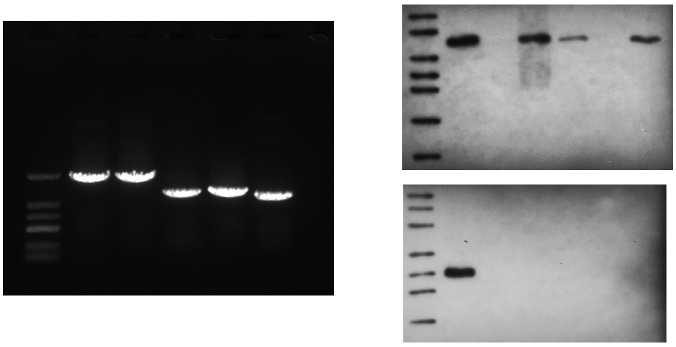

4F

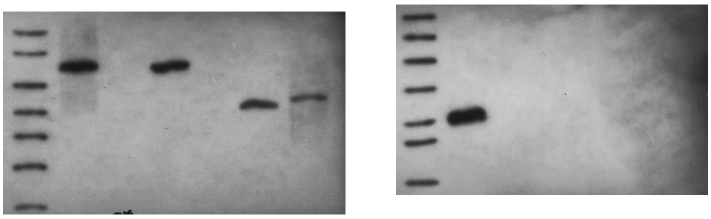

4G

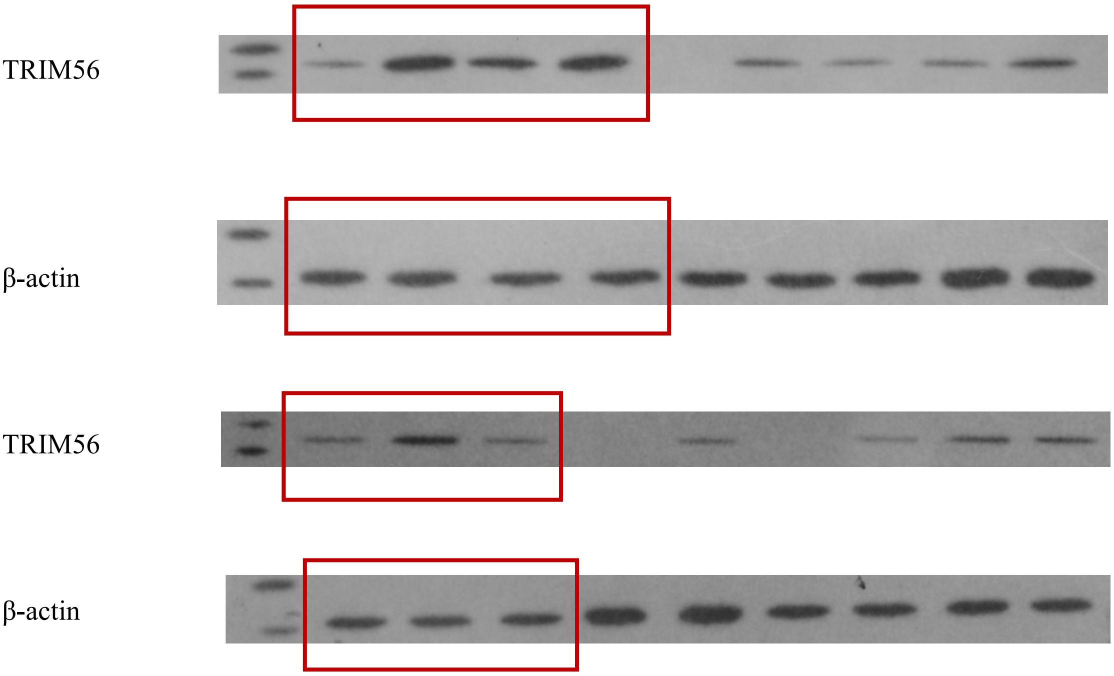

4H

TRIM56

β-actin

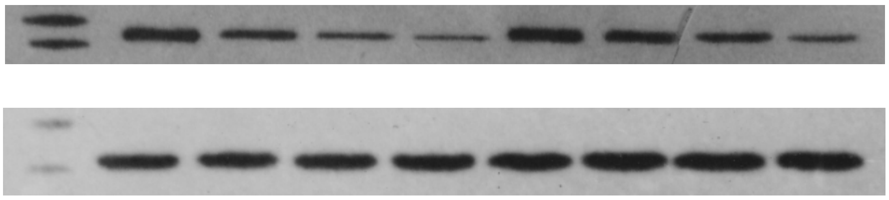

4I

TFAM

PGC-1α

CV-ATP5A

CIII-UQCRC2

CIV-MTCO1

CII-SDHB

CI-NDUFB8

Bax

β-actin

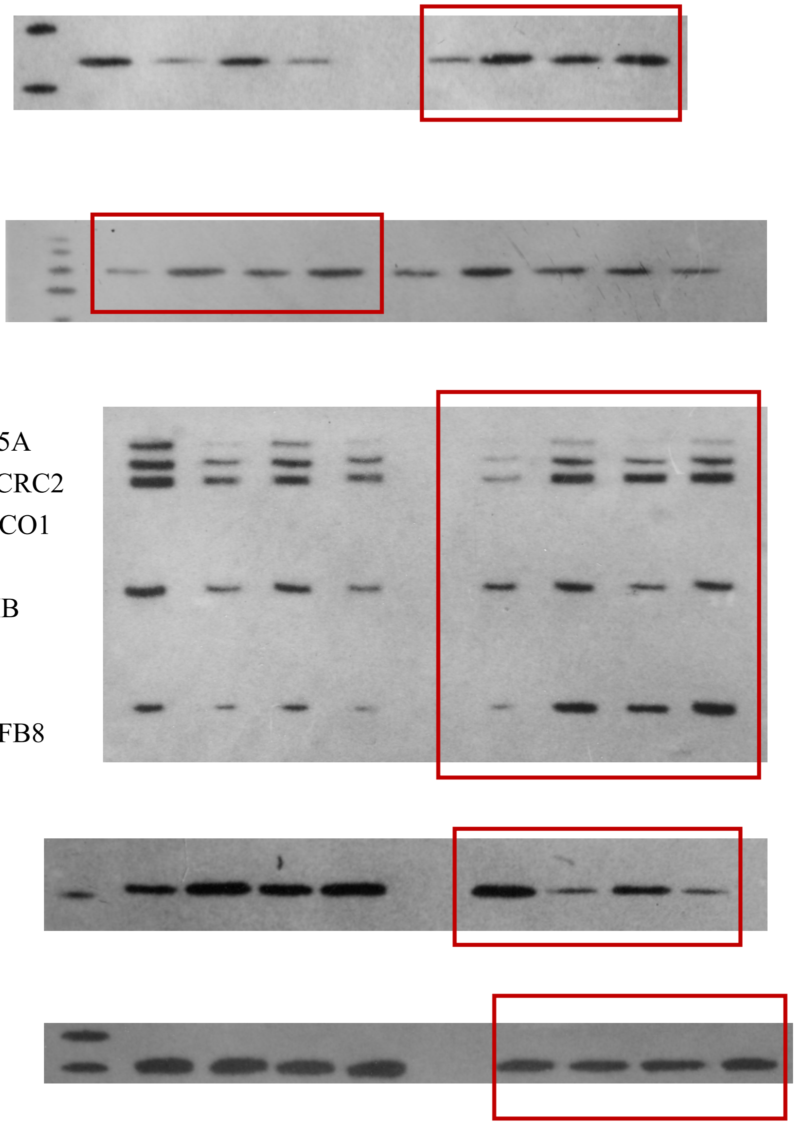

4N

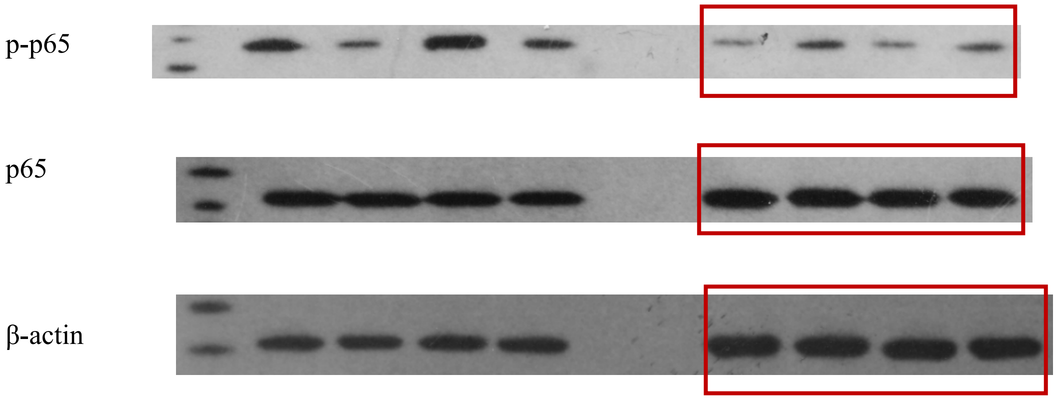

Figure 5

5A

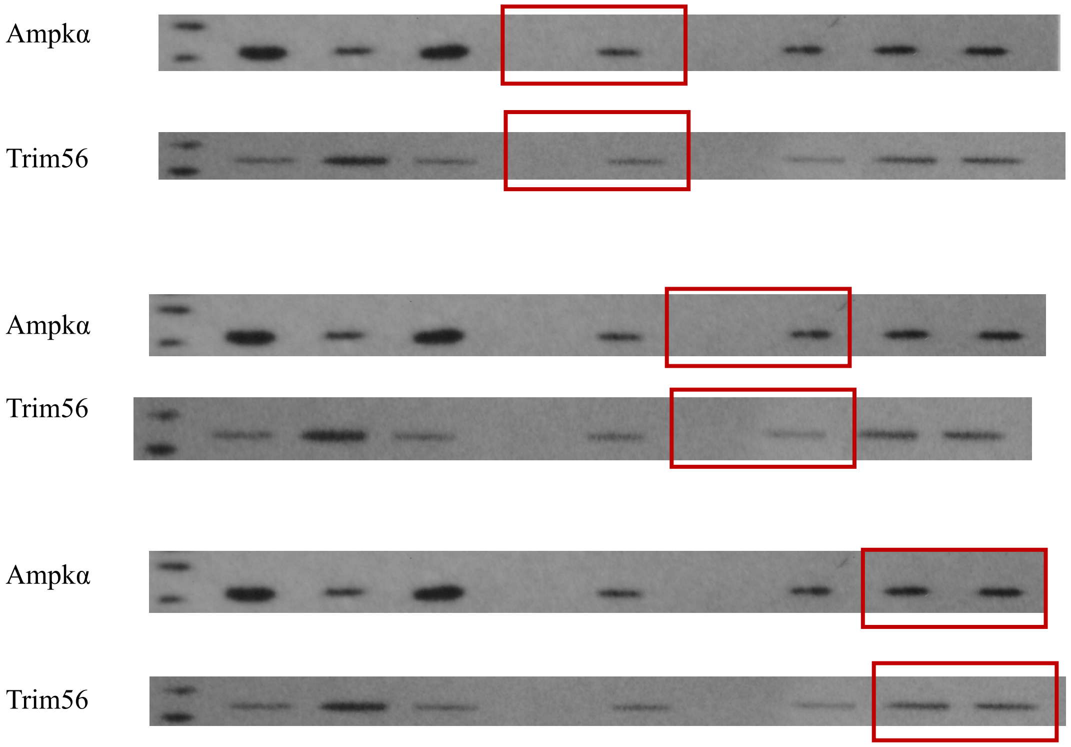

5C

Ampkα

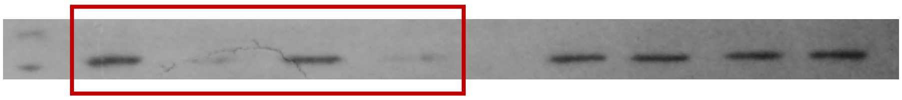

β-actin

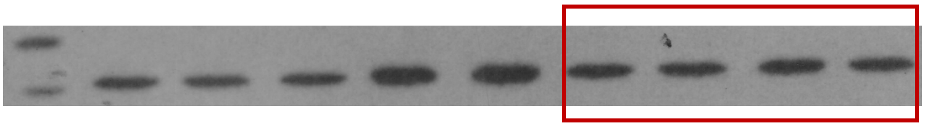

5D

Ampkα

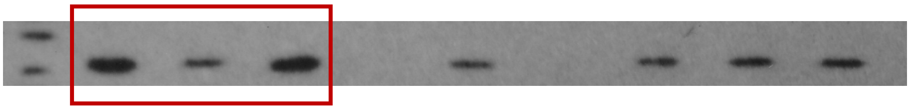

β-actin

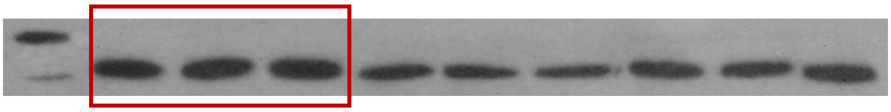

5E

Ampkα

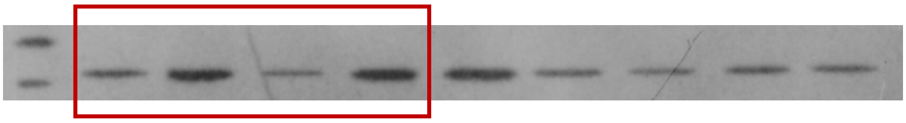

β-actin

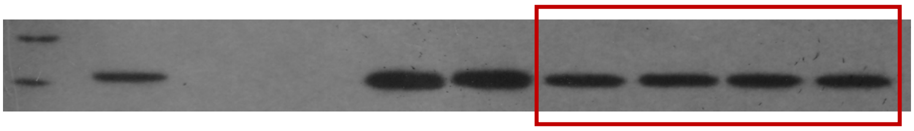

5F

Ampkα

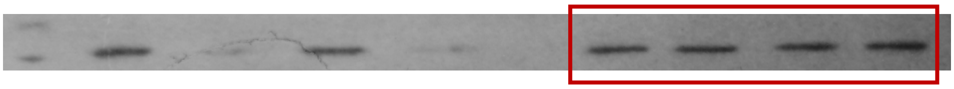

Trim56

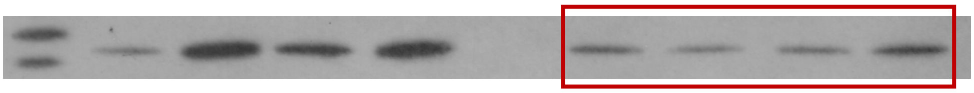

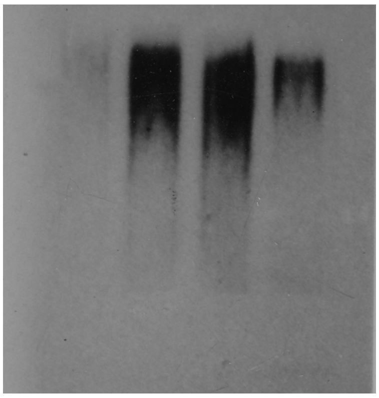

5G

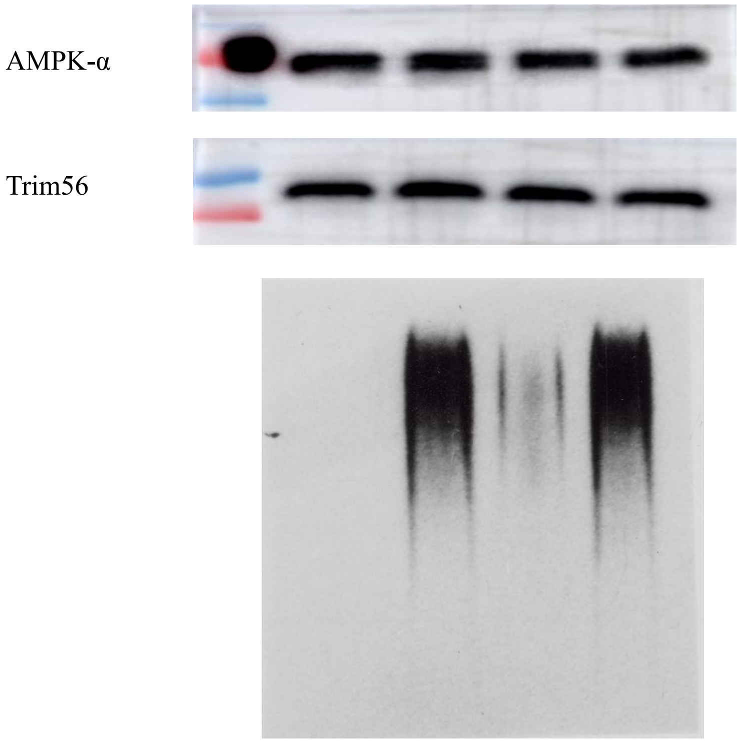

5H

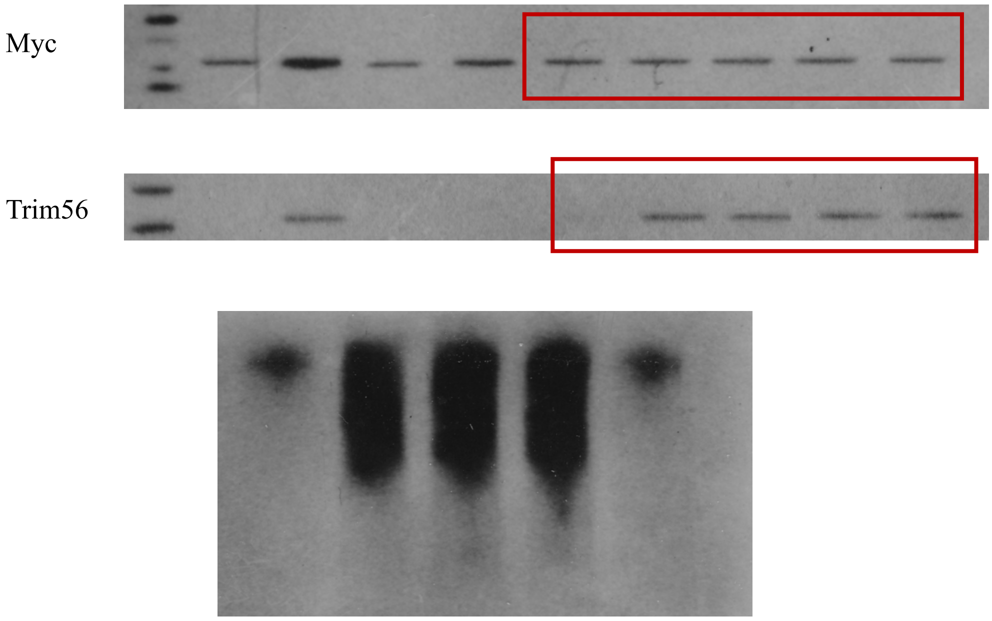

Figure 6

6A

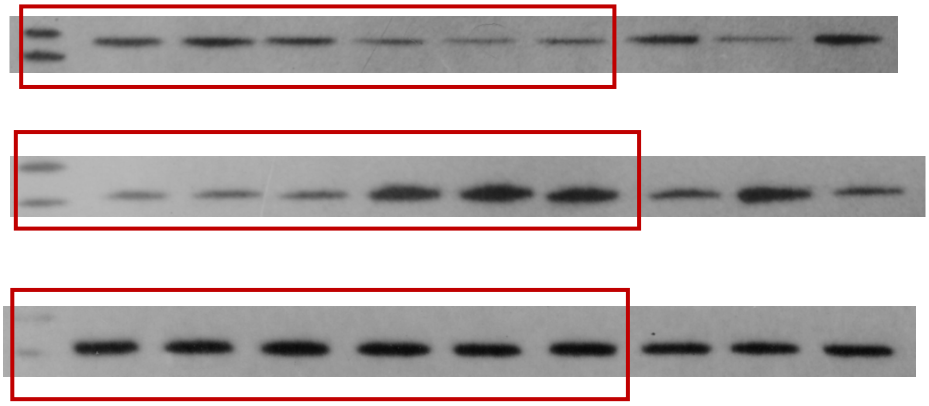

Trim56

AMPKα

Actin

Figure 7

7E

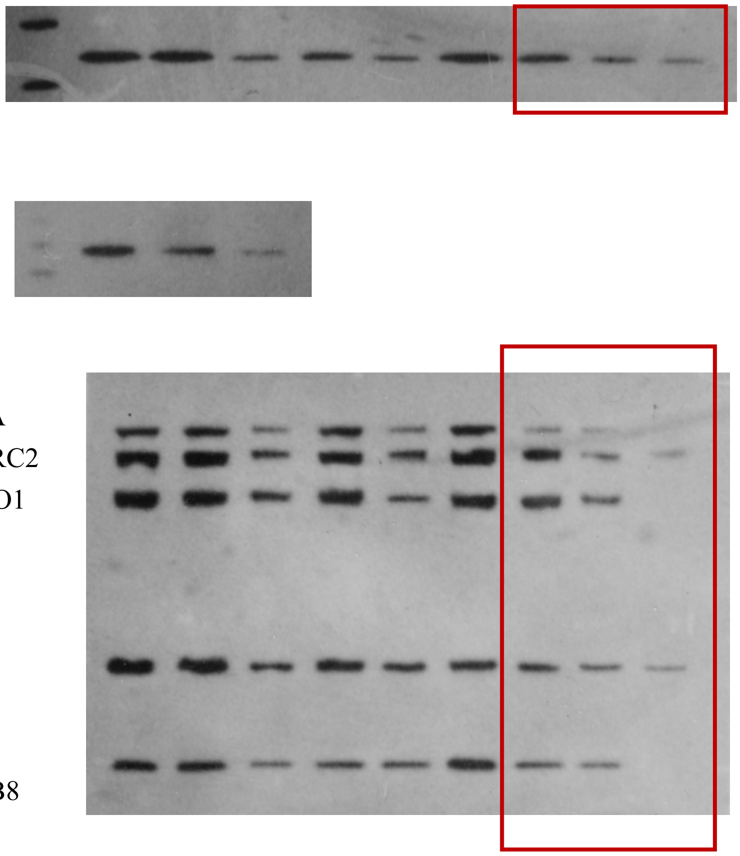

TFAM

PGC-1α

CV-ATP5A
CIII-UQCRC2
CIV-MTCO1

CII-SDHB

CI-NDUFB8

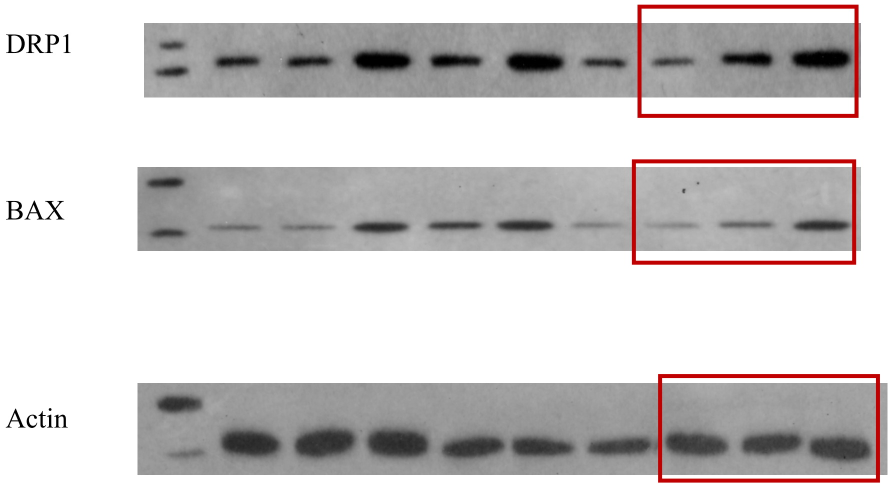

7F

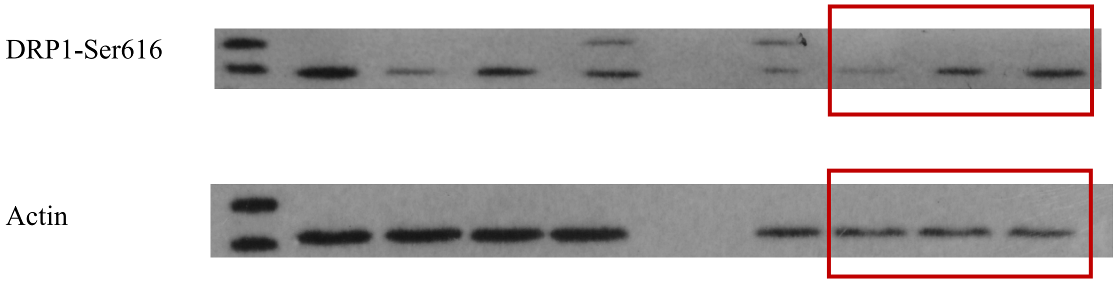

7N

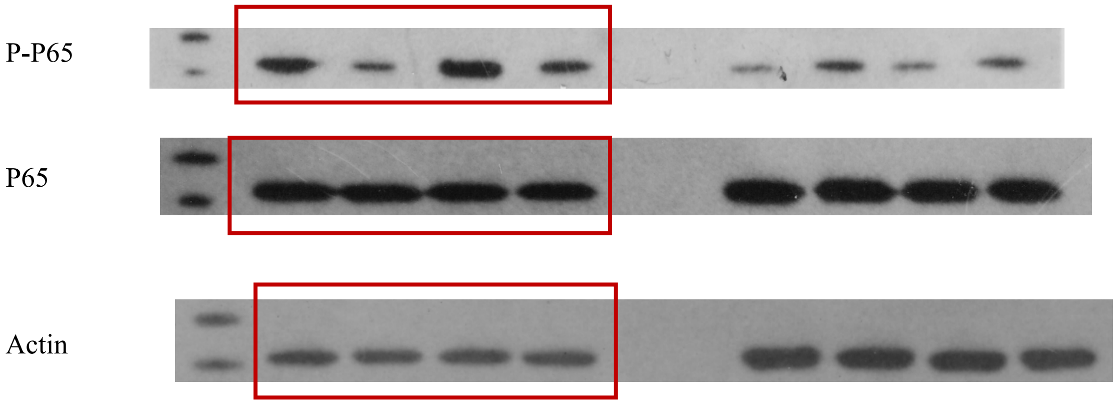

FigureS2

S2B

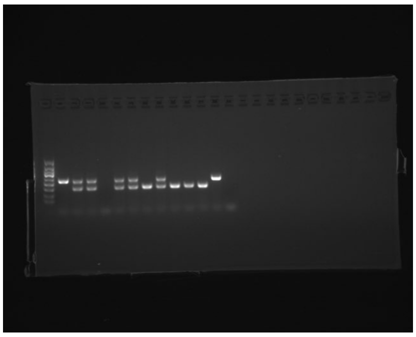

Figure S4

S4C

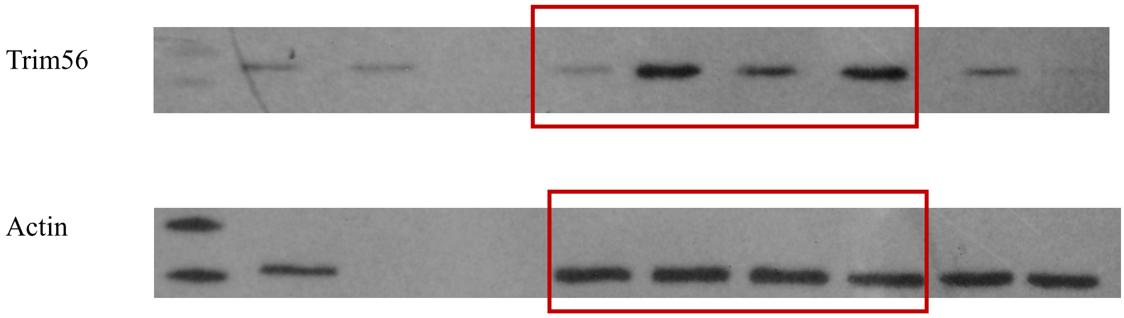

S4D

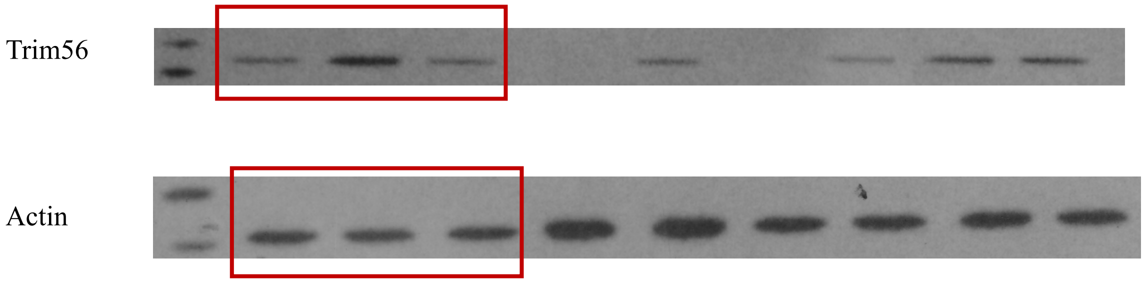

FigureS5B

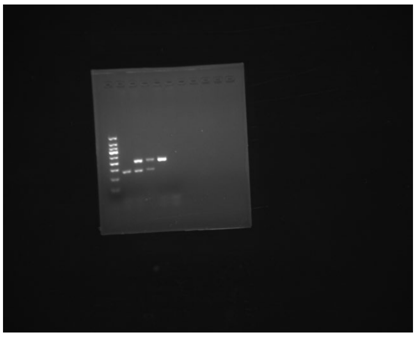

FigureS6

S6A

ampkα

Actin

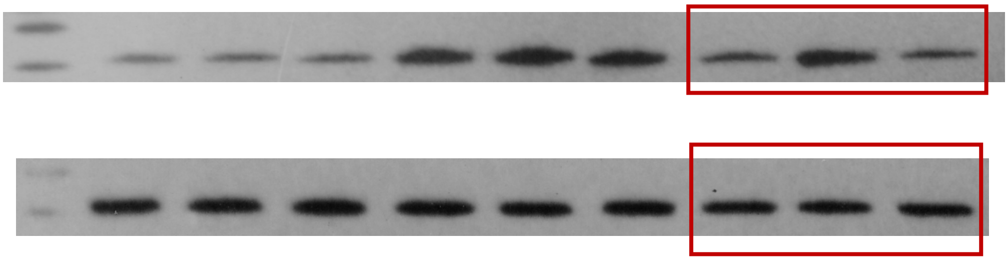

S6D

ampkα

Actin

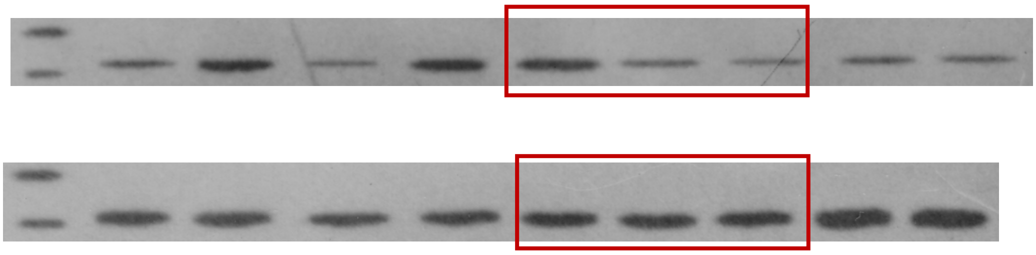

S6F

ampkα

Actin

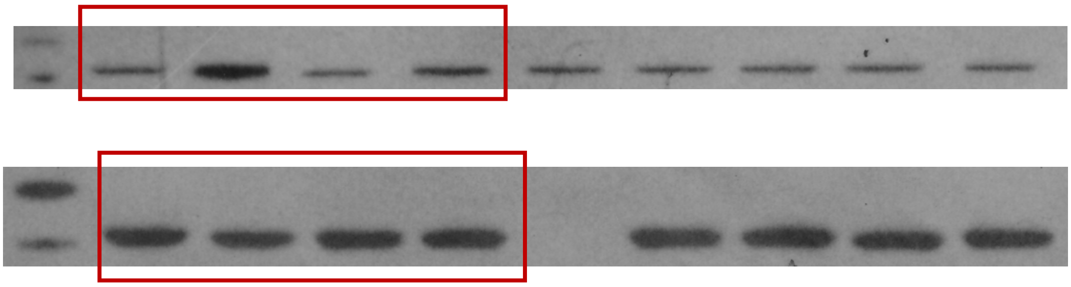

Supplement: Supplementary file 1 — Supplementary Materials [file 41419_2024_7107_MOESM1_ESM.pdf]
